# Effect of Resource Abundance on Woodland Rodents' Demography at Latitudinal Extremes in Europe

**DOI:** 10.1002/ece3.71466

**Published:** 2025-06-02

**Authors:** Giulia Ferrari, Olivier Devineau, Valentina Tagliapietra, Kaja Johnsen, Federico Ossi, Francesca Cagnacci

**Affiliations:** ^1^ Inland Norway University of Applied Sciences, Faculty of Applied Ecology, Agricultural Sciences and Biotechnology Koppang Norway; ^2^ Fondazione Edmund Mach Research and Innovation Centre San Michele all'Adige (Trento) – Italy; ^3^ NBFC, National Biodiversity Future Center Palermo Italy

**Keywords:** demography, mast‐driven outbreaks, population cycles, supplemental feeding, woodland rodents

## Abstract

Climate change effects on primary productivity are especially evident along altitudinal and latitudinal gradients. Some of the species with a fast reproductive cycle strategy and relying on primary productivity may rapidly respond to such changes with alterations to demographic parameters. However, how these bottom‐up effects may emerge in systems with different population dynamics has not been elucidated. We aimed to assess the role of food availability on rodent demography in populations characterised by different dynamics, that is multiannual cycles in Northern European populations and mast‐driven outbreaks in Southern European populations, both driven by intrinsic and extrinsic factors. We live‐trapped woodland rodents at these latitudinal extremes in two study systems (Norway, Italy) while deploying control/treatment designs of food manipulation providing ad libitum trophic resource availability, albeit not reflecting the natural resource fluctuations. We applied a multistate open robust design model to estimate population patterns and survival rates while controlling for seasonal variation, intrinsic traits, and co‐occurrence of sympatric species. Yellow‐necked and wood mouse (*Apodemus* spp.) were sympatric with bank vole ( *Clethrionomys glareolus* ) in Italy, while only the latter was trapped in Norway. Food provisioning increased both survival and population size of bank vole in Norway, where temperatures are harsher and snow cover persists in winter. In milder Italian habitats, the wood mouse abundance was boosted by food availability, increasing also survival rates (but only in females), whereas the bank vole showed a decrease in both parameters across sexes. We speculate that overabundant food resources may trigger some forms of competition between sympatric wood mouse and bank vole, although other types of interactions, such as predation and parasitism, may also contribute. By manipulating food availability in two systems where rodents have different population dynamics, we showed how resource availability exerted bottom‐up effects on rodent demography, especially in the context of climate change, although being mediated by other intrinsic and extrinsic factors.

## Graphical abstract

The study aimed to assess the role of food availability on rodent demography in populations characterised by different dynamics, that is multiannual cycles in Northern European populations and mast‐driven outbreaks in Southern European populations.

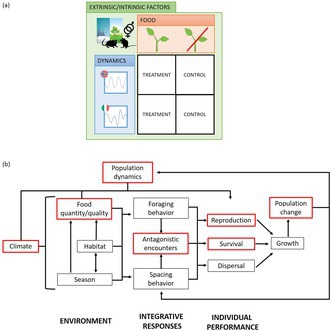

## 1 Introduction

Populations of most species exhibit spatial and temporal variations in size that define their population dynamics. Predicting how populations respond to environmental pressures is crucial for effective wildlife management and conservation strategies to maintain biodiversity. Multiple factors, both intrinsic and extrinsic, intervene and interact with each other to govern the population dynamics of mammals (Erb et al. 2001), with natural variability modulating synchronous or divergent patterns across space and time. Specifically, intrinsic factors (i.e., density dependence, behaviour, physiology and life‐history traits) are more likely to cause year‐round variations in survival and reproduction (Aars and Ims 2002), while extrinsic environmental conditions, such as climatic constraints (Johnsen et al. 2018) and food availability (Boutin 1990; Soininen and Neby 2024), interact with intrinsic factors to trigger seasonal changes in population demography. For instance, rodent population dynamics are determined by both direct (first‐order) and delayed (second‐order) density dependence that influence growth rates by current and past population density, respectively (Stenseth 1999). Direct density dependence may induce population regulation through an immediate negative feedback from population density to growth rates (Inchausti et al. 2009), for example, through social mechanisms or territorial behaviour that increase competition for resources. Instead, delayed density dependence implies a change in growth rates due to specialist predators, plant‐herbivore interaction that decreases food quality, or pathogens responding to past density (Oli 2019). Density dependence can generate a variety of dynamical population patterns across ecosystems, regulating population size and stability by interacting with extrinsic environmental factors. Also, inverse density dependence, termed Allee effects (Allee 1927; Stephens and Sutherland 1999), may emerge in structuring population dynamics and is defined as the positive effects of increasing density on fitness (i.e., growth rate). Allee effects have a predicted consequence on multiple mechanisms in small or low‐density populations and are expected to affect many population‐level processes, including extinction (Berec et al. 2007; Kramer et al. 2009).

Small rodent species studies have often contributed insights into the ultimate causes shaping population dynamics in wild populations. This is because, thanks to their r‐selection strategy and their rapid functional and numerical responses to climatic and environmental changes (Zwolak, Bogdziewicz, Wróbel, et al. 2016), these species can provide multi‐generational observational data in a short time period, even in wild settings (Krebs 1988; Lambin and Krebs 2002; Andreassen et al. 2020). Demographic mechanisms, including density dependence, often emerge within a community of species, where interspecific interactions, such as competition, predation, and parasitism, occur (Huitu et al. 2004; Radchuk et al. 2016) and vary across different geographic regions, seasons, and among species (Andreassen et al. 2021). Indeed, small rodents are characterised by erratic population patterns, which can either occur through extreme eruptive dynamics or show regular multiannual fluctuations. In particular, in the northern latitudes where density dependence weakens, grass‐eating voles (*Microtus* spp., *Clethionomys rufocanus* and *C. rutilus* ) and lemmings ( *Lemmus lemmus* ) exhibit periodic multiannual 4‐phase cycles (i.e., increase, peak, decrease, crash) characterised by wide amplitude between minimum and maximum densities and peaks every 3–5 years (Krebs 2013; Radchuk et al. 2016; Johnsen, Devineau, et al. 2019). Elsewhere, populations of voles (*M. subterraneous*, *C. glareolus* ) and woodland mice (*Apodemus* spp.), as well as African rodents ( *Mastomys natalensis* ), are typically characterised by large outbreaks with irregular intervals, usually coupled with plant seed production and pulsed resources (e.g., mast seeding) (Leirs et al. 1997; Zwolak et al. 2018; Selås 2020; Suzuki et al. 2022). Within these two contrasting frameworks, predation can shape amplitude and period cycle of rodent population dynamics, affecting growth rate and annual recovery (Andreassen et al. 2020), while also influencing dispersal and foraging efficiency (Puig‐Gironès 2023). Changes in rodent populations can also alter intra‐ and inter‐specific competition, largely based on exploitation competition and interference competition that operate limiting resource allocation and habitat use, respectively (Eccard and Ylönen 2003).

Sinclair and Krebs (2002) generalised that food supply is the primary factor determining animal population growth rate (bottom‐up control), and only secondarily it is overridden or severely modified by regulatory top‐down processes (predators), social interactions, and stochastic disturbance. However, in small rodents, this single‐factor hypothesis, also cautiously postulated by Turchin and Batzli (2001), has been rapidly replaced with a more comprehensive multi‐factorial explanation, intended as an interplay between top‐down and bottom‐up mechanisms driving changes in population dynamics (Krebs 2013). More recently, Flowerdew et al. (2017) supported the earlier hypothesis, showing that the demography of *C. glareolus* and *A. sylvaticus* was strongly influenced by bottom‐up food availability and density dependence, being only weakly mediated by seasonality and predation. This controversy emerged also when considering the effect of food resources on single demographic parameters of rodents at an intra‐annual scale (e.g., density, reproduction, and survival). Several studies used food supplementation to experimentally test these effects, finding that supplemental food favored breeding in *M. californicus* (Krebs and DeLong 1965), increased reproduction in *M. pennsylvaticus* (Desy and Thompson 1983) and promoted survival in *C. glareolus* (Johnsen et al. 2017), so leading to an increase in population density (Cole and Batzli 1978; Taitt and Krebs 1981; Yoccoz et al. 2001). In contrast, other studies observed that food supply decreased survival in *Peromyscus leucopus* (Hansen and Batzli 1978) and population density of *M. californicus* (Krebs and DeLong 1965), as well as growth rate in *C. glareolus* (Löfgren et al. 1996). These contrasting findings also point at density dependence effects. While overgrazing may trigger delayed density dependence on herbivore populations via herbivory‐induced reductions in plant quality (Krebs et al. 2010; Reynolds et al. 2012; read also Ruffino et al. 2018), food supply generally influences population demography with direct density dependence, by affecting survival and reproduction (Huitu et al. 2003). In the presence of exogenous temporal variations of food availability, for example, during abundant seed productivity (i.e., masting), an increase in demographic parameters may boost population density to carrying capacity, thus inducing a successive delayed rebound (Lobo and Millar 2013; Rossi and Leiner 2022, 2024). Yet, the complex and variable. Hence, the effect of the availability of food resources on small rodents' demographic patterns merits further investigation.

The different population dynamics patterns expressed by small rodents along the latitudinal gradient can modulate the demographic responses of these species to food availability. In northern populations undergoing multiannual cycles, the effect of temporal variation in food availability on rodent demography is not fully clarified (Huitu et al. 2003; Johnsen et al. 2017; Oli 2019), although food supply does not seem to prevent such cycles from occurring (Krebs 2013) but is identified as a direct density‐dependent limiting factor (Huitu et al. 2003). Conversely, in temperate forest systems where climate is not a limiting factor and mass events of seed production occur (or masting, a seed‐predator avoidance strategy evolved by plants according to the predator satiation hypothesis, Vacchiano et al. 2018; Zwolak et al. 2022), the increment of food availability enhances habitat quality and, ultimately, when habitat structure is also adequate, its carrying capacity. Specifically, the occasional boost of food availability induces lagged eruptive demographic outbreaks first (functional response), supported by higher survival rates, increased reproductive success, and enhanced juvenile recruitment. However, then rodent population crashes (numerical response) due to limiting food resources and a potentially increased predation pressure dampening growth rates (Ostfeld and Keesing 2000; Bogdziewicz et al. 2016). This occurs mainly in granivorous rodent species whose diet is based on seeds and floral parts (e.g., woodland mice), while it moderately affects generalist species with an intermediate trophic position, such as voles, which can find other food resources. Demographic responses of small rodents to pulsed seed production are also mediated by other environmental factors, such as local climatic conditions, geographical location, and habitat fragmentation, resulting in heterogeneous demographic responses, which in turn shape rodent inter‐specific relationships with other vertebrate species (Wittmer et al. 2007; Zwolak et al. 2018). The relation between food availability and rodent demography is further complicated by the fast ongoing climate change that leads to alterations in plant primary production and nutritional quality, with changes in vegetation composition and distribution. For instance, climate change is affecting inter‐annual variation and synchrony in seed production across altitudinal and latitudinal gradients (Bogdziewicz et al. 2021; Hacket‐Pain and Bogdziewicz 2021), with masting events becoming more frequent or occurring at higher latitudes than today (Pearse et al. 2020). These climate‐driven changes have unavoidable consequences for herbivorous small rodents across different geographic regions (Puig‐ Gironès et al. 2023; Soininen and Neby 2024). Indeed, voles and mice may experience changes in population cycles (Cornulier et al. 2013; Suzuki et al. 2022), a decline in the abundance of cryptic species (Torre et al. 2023) and distributional shifts (Myers et al. 2009).

**FIGURE 1:**
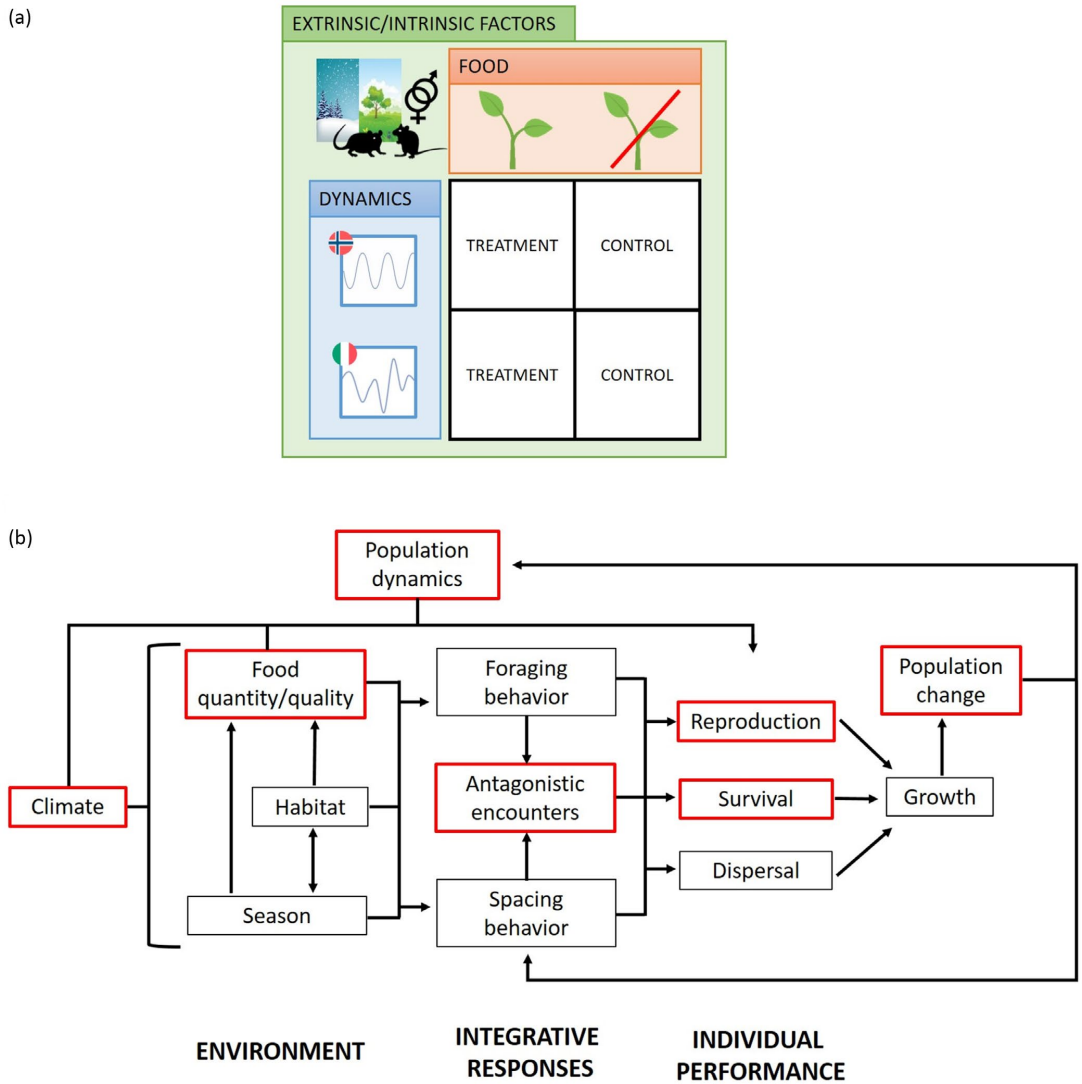
Panel (a) – Schematic representation of the experimental design. Based on treatment‐control designs of food supplementation (in orange), we compared rodent live‐trapping at two latitudinal extremes (Norway and Italy) where rodents have different population dynamics, that is cycles and mast‐driven outbreaks, respectively (in blue). We also controlled for seasonal variation, individual traits, like sex, and sympatric species co‐occurrence (in green). Panel (b) – Scheme of the relationships between environment, integrative responses and individual performances occurring in small rodents (readapted from Batzli 1992). The red boxes indicate the cause‐and‐effect relationships addressed in this study. Specifically, the experimental manipulations permitted to evaluate individual performance (survival, reproduction) and population abundance change in dependence on food availability under contrasting population dynamics patterns, while accounting for climate harshness and species co‐occurrence.

In this context, our study aimed to evaluate changes in woodland rodent demography parameters (i.e., survival and population size) in relation to food availability, which we manipulated along the cyclic‐eruptive continuum of small rodents' dynamics. In particular, we performed a descriptive comparison of two independent study systems, which are based on food manipulations performed at latitudinal extremes of the boreal‐temperate gradient (Figure 1), that is South‐East Norway (Johnsen et al. 2017) and the Italian Alps (Ferrari 2022). In these two systems, small rodents exhibit multiannual periodic cycles in Scandinavia (Johnsen, Devineau, et al. 2019) or eruptive and irregular outbreaks associated with mast events at lower latitudes (Marini, Tagliapietra, et al. 2023). Despite the heterogeneity that emerged between the two systems, we aimed to investigate the relationship between food availability and demographic parameters by qualitatively interpreting the results within the same theoretical framework, taking into account the differences across sampling design when defining the analyses. In both study systems, woodland rodents were intensively live‐trapped, and food resource availability was manipulated in wild settings, by providing artificial supplemental feeding in treatment/control designs, while also seasonal variation, individual traits and occurrence of sympatric species were recorded (Figure 1a). We hypothesised that food supply would not necessarily alter demographic parameters in Norwegian cyclic populations, while the demographic parameters of Italian rodents' mast‐driven populations would be more heavily affected by the provisioning of food (H1). We also hypothesised that seasonality and climate harshness on the one side (Andreassen et al. 2020; H2.1), individual traits such as sex (H2.2) and the co‐occurrence of other woodland species on the other (H2.3) might locally alter these expected general patterns characterising the demography of rodent populations under investigation (Figure 1b).

## 2 Materials and Methods

### 2.1 Study Areas

**FIGURE 2:**
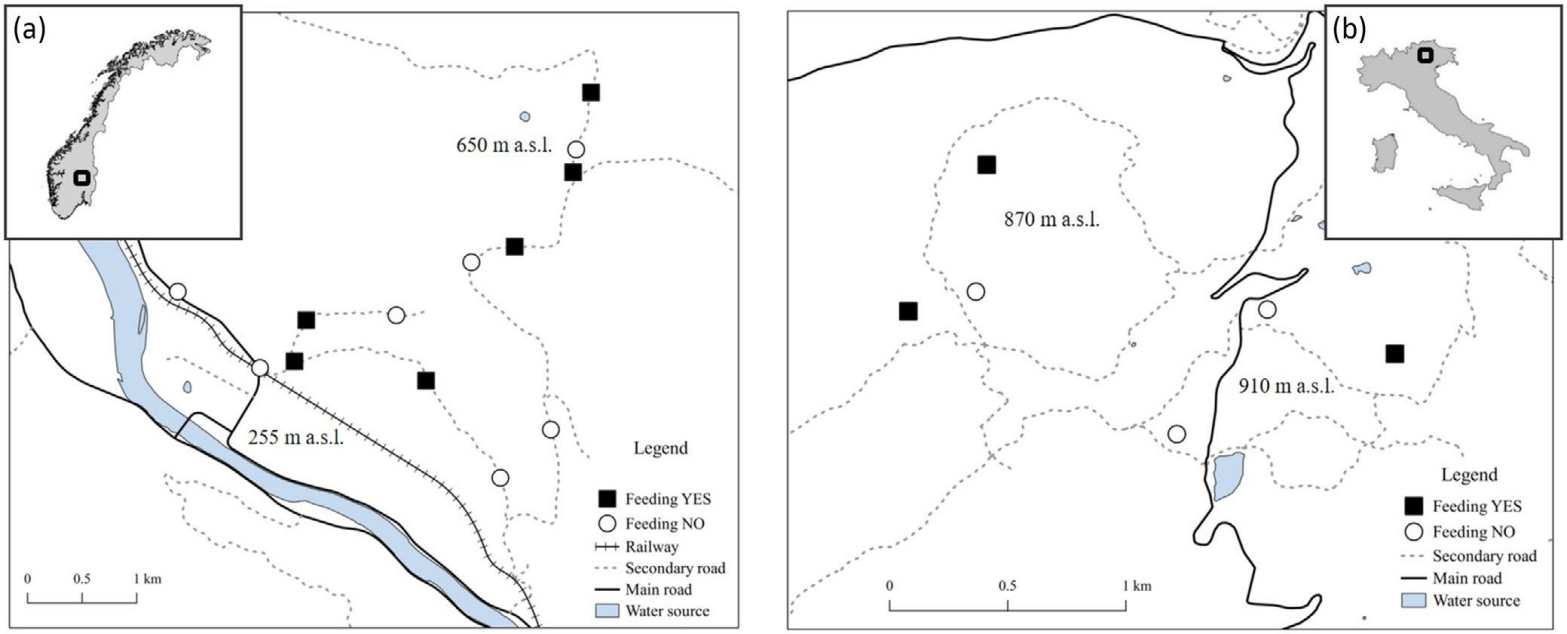
Maps and locations of the study areas. (A) Evenstad area, in South‐Eastern Norway (61.00 N—11.00E), monitoring period: 2013–2015; (B) Cembra area, in North‐Eastern Italian Alps (46.13 N—11.17E), monitoring period: 2019–2021. In both study areas, rodents were live‐trapped at sites with (black squares) and without (white circles) supplemental food.

The Norwegian study was performed in the proximity of Evenstad (61.00 N—11.00E; Stor Evdal municipality; Figure 2A) and includes the bottom of the valley of the Glomma river basin and the surrounding hills (255–700 m a.s.l.), but excludes the agricultural open plains. The study area is characterised by a boreal climate (Dfc: no dry season, cool winters and summers; *sensu* Köppen‐Geiger classification, Kottek et al. 2006), with long, harsh, and snowy winters with a permanent snow layer (from December to April) and short, cool summers. Mean daily temperatures were −8.9°C ± 3.2°C in January and 14.2°C ± 2.1°C in July, and mean monthly precipitation was 35.2 ± 14.7 mm in January and 84.1 ± 40.4 mm in July (data from the Norwegian Meteorological Institute https://seklima.met.no/, Evenstad, Åkrestrømmen, Drevsjø and Gløtvola weather stations at 260/670 m a.s.l., period 2000–2020). The landscape is dominated by a homogenous boreal coniferous forest of *Picea abies* and *Pinus sylvestris* , bilberry ( *Vaccinium myrtillus* ) in the understory shrub layer, and mosses (e.g., *Pleurozium schreberi* ) on the ground layer (Johnsen et al. 2017). In this study site, the community of mammals covers boreal taxa, including forest ungulates, such as *Alces alces* , *Cervus elaphus* , *Rangifer tarandus* , and *Capreolus capreolus* , as well as carnivores (e.g., *Canis lupus* , *Vulpes vulpes* , *Gulo gulo* , *Meles meles* , *Lutra lutra* , *Ursus arctos* , *Linx linx* and several *Martes* and *Mustela* species) that retain a crucial role in prey–predator dynamics involving small mammals (Korpela et al. 2014). Among small mammals, insectivores (*Sorex* spp., *Erinaceus europaeus* , *Neomys* spp.) and most of *Cricetidae* ( *Clethrionomys glareolus* and *C. rufocanus* , *Lemmus lemmus* , *Myopus schisticolor* ) occupy forested areas, while *Muridae* ( *Apodemus flavicollis* and *A. sylvaticus* ) together with *Microtus* spp. occur in agricultural plains (Chester 2016).

The Italian study was located in Cembra Valley (46.13 N—11.17E; Autonomous Province of Trento; Figure 2B), and the study area is characterized by moderate topography (500–1000 m a.s.l.) in orographic continuity with the Lagorai massif. It is characterised by warm‐temperate climate (Cfc class: no dry season, cool summers; *sensu* Köppen‐Geiger classification, Rubel et al. 2017), with moderately cold winters, occasional snow cover and usually frozen ground, and fresh summers. Mean daily temperatures were 0.9°C ± 1.3°C in January and 20.8°C ± 1.4°C in July, and mean monthly precipitations were 39.0 ± 1.3 mm in January and 112.9 ± 47.8 mm in July, which were recorded (data from Meteotrentino https://www.meteotrentino.it, Cembra weather station at 652 m a.s.l.; period 2000–2020). The area is almost entirely covered by relatively homogeneous secondary growth mixed forest, dominated by *Pinus sylvestris* with abundant shrub undergrowth, as well as mixed stands of *Fagus sylvatica* , *Picea abies, Abies alba* and, to a lower extent, *Quercus petraea* , interspersed with peat bogs and small pastures. In 2020, a mast seeding event of both *Fagus sylvatica* and *Picea abies* occurred in the region (authors' pers. obs., also recorded in Marini, Arnoldi, et al. (2023) in a similar area of the Autonomous Province of Trento). The mammalian community in the area accounts for a variety of taxa, including forest ungulates ( *C. capreolus* , *C. elaphus* , *Sus scrofa* ), as well as carnivores ( *C. lupus* , *V. vulpes* , and several mustelids, such as *Martes* spp. and *Mustela* spp. and *M. meles* ). Both ungulates and carnivores can affect small mammal populations through foraging activity (Mori et al. 2020) and predatory control (Mortelliti et al. 2009), respectively. Among small mammals, the community includes two main groups, beyond *Chiroptera*: insectivores (e.g., *Sorex* spp., *Neomys* spp., *Crocidura* spp., *Talpa* spp. and *Erinaceus* spp.) and woodland rodents, including *Gliridae* and *Sciuridae* ( *Sciurus vulgaris* , *Glis glis* , *Muscardinus avellanarious*, *Elyomis quercinus*), *Cricetidae* ( *C. glareolus* , *Microtus* spp.) and *Muridae* ( *A. flavicollis* and *A. sylvaticus* ) (Deflorian et al. 2018).

Previous studies ascertained that the common vole species between the two study areas (i.e., *C. glareolus* ) showed opposite patterns of population dynamics. In particular, these woodland‐dwelling voles experience regular 4‐year cycles driven by trophic interactions and delayed density dependence at the Evenstad study area (Johnsen, Devineau, et al. 2019; Andreassen et al. 2020), while they exhibit irregular outbreaks determined by bottom‐up trophic effects of seed mast combined with direct density dependence in Cembra Valley (Marini, Tagliapietra, et al. 2023).

### 2.2 Experimental Design

In the two study systems, woodland rodent populations were exposed to feeding experimental manipulations, aiming at responding to different research questions. In particular, in Norway Johnsen et al. (2017) examined the role of winter conditions, habitat structure, and food availability as potential causes for winter population crashes, while in Italy Ferrari (2022) considered the implications of anthropogenic ungulate feeding sites on host–parasite‐pathogen dynamics and disease risk. Despite differences in experimental designs (e.g., food type, grid pattern, and spatial distribution of food), both studies relied on rodent live‐trapping and control/treatment feeding experiment consisting in providing ad libitum supplemental food in some trapping occasions and/or sites (‘Feeding yes’), but not in others (‘Feeding no’). Here, we leveraged these manipulation occurrences to independently evaluate and descriptively compare the role of food resources on rodent demography, accounting for climatic and species occurrence differences (Figure 1).

Specifically, in Norway, we deployed thirteen cross‐shaped trapping grids of 16 traps each (total traps: 208; total grid area: 3600 m^2^; see Appendix S1 for further details) and performed monthly captures between 2013 and 2015 (see Appendix S2, Table S2.1 for a summary of capture sessions). Captures lasted 4 days (i.e., 3 nights) per month, and traps were checked every 12 h to prevent nocturnal mortality due to harsh climate (for additional information on the protocol in Norway, see Johnsen et al. 2017). In six of those grids, supplemental food (a mixture of oat and sunflowers) was provided ad libitum next to each trap from December 2013 to June 2014, in November 2014, and in May 2015. In these six grids from June to August 2015, and in the remaining seven grids from December 2013 to August 2015, no food was provided. Each trap was covered with a 30 × 30 × 40 cm floorless plywood box to prevent the traps from being covered with snow (Johnsen et al. 2017). This also limited access to large non‐target species to the supplemental ad libitum food, although possibly used by birds and *S. vulgaris* .

In Italy, we deployed six squared 70 × 70 m trapping grids of 64 traps each (total traps: 384; total grid area: 4900 m^2^; see Appendix S1 for further details) and performed monthly captures from November to March and bi‐monthly captures in April, June, and August, between February 2019 and April 2021 (see Appendix S2, Table S2.2). Captures lasted 4 days (i.e., 3 nights) per month or bi‐month, and traps were checked every 24 h. Three of these grids were always provided with ad libitum corn supply dispensed through ungulate supplemental feeding stations located at the centre of each grid, while the other three were located at least 500 m from any feeding site. Food supplementation at feeding sites in Italy had been persisting for several years at the time of our study. Although the feeding stations were also visited by other non‐target species (e.g., *C. capreolus* , *M. meles* , *S. vulgaris* and several bird species), the supplemental food was provided ad libitum.

We also controlled for (i) seasonal variation; (ii) individual traits and (ii) sympatric species co‐occurrence. Given the intra‐annual growth activity of Alpine temperate forests (April–October; Etzold et al. 2022) and the range of the growing season in southern parts of Norway (mid‐April—October; Skaugen and Tveito 2004), we decided to differentiate the entire sampling period into two seasonal periods identified as crucial for small rodent life history across latitudes (Eccard and Herde 2013). Specifically, we aggregated months into favorable (namely ‘summer’: April–October) and limiting (namely ‘winter’: November–March) seasonal periods, for both study areas. Moreover, we evaluated the effect of sex on demographic parameters, as it can affect survival and reproductive success, as well as spacing behaviors (Rémy et al. 2013). Furthermore, where sympatric rodent species occurred (i.e., where the overlap between voles and woodland mice occurs), we independently assessed demographic parameters and compared their patterns. Finally, we tested for potential inter‐specific competition effects (see par. 2.3).

In both the Italian and Norwegian study areas, the trapping grids were placed at least 500 m from each other to ensure spatial independence across grids. The traps were left open during non‐trapping periods to habituate animals to their presence. Each trap consisted of a standard Ugglan Multiple Live Trap (model 2, Granhab, Sweden; Jung 2016), filled with hay during the cold season and baited with carrot slices and seeds (oat in Norway, sunflower in Italy). Standard capture‐mark‐recapture (CMR) techniques were adopted (Amstrup et al. 2005; Pollock et al. 1990). Each animal was individually marked with a Passive Integrated Transponder (PIT) tag (Trovan Ltd., UK). Standard information (date, time, trap number, grid number, ID of the animal) and life‐history traits (species, sex, body mass, age, breeding status) were recorded for each capture.

All animal handling procedures and ethical issues were approved by the relevant regional or local Wildlife Management Committees.

### 2.3 Multistate Open Robust Design Models and Statistical Analyses

We analysed capture data separately for Norway and Italy to account for the differences in the experimental design (see par 2.2 *Experimental design*).

Our field protocol, defined by daily secondary occasions within monthly primary ones, corresponds to the so‐called robust design framework (Pollock 1982). Therefore, we used a multistate open robust design (MSORD) approach to estimate demographic parameters (Cooch and White 2019; White et al. 2006). This model provides five parameters (Kendall and Nichols 2002; Boys et al. 2019; Cooch and White 2019): (i) the apparent survival (S_t_) hereafter ‘survival’, which expresses the probability of surviving from release occasion *t* to subsequent primary occasion *t* +1 occupying a state s; (ii) transition probability (*ψ*_s_), which represents the probability of animals transitioning to different states s between primary occasions *t* and t + 1; (iii) entry or arrival probability (*pent*_*j*_), which expresses the probability of an animal arriving and being captured in the study area in secondary occasion j; (iv) persistence probability (*φ*_*j*_), which is the probability of an individual to survive and persist in a capture site at secondary occasion j, given it was present in j—1; (v) capture probability (*p*_*j*_), the probability of an animal being detected at occasion j, given it was present. The transition probability (*ψ*) was intended differently for Norway and Italy, given the different spatio‐temporal distribution of supplemental feeding performed in the two study systems. For the former, it consisted in the probability of transition between states of food availability at trapping sites, expressed by the covariate ‘feeding state’, as feeding was treated as a spatio‐temporal covariate (i.e., availability of food in certain primary occasions in certain sites; see also 2.2 *Experimental design*); for the latter, it regarded the probability of transition between state of individual observability, that is animals being present in the study area and available for detection (observable state) or otherwise temporary emigrant outside the study area (unobservable state), and it was set as constant. The transience between states in both Italy and Norway allowed to consider the emigration of transient animals, as well as the spatio‐temporal variability of food. The availability of supplemental food for Italy was expressed instead by a spatial, temporally fixed covariate, ‘feeding site’, corresponding to the trapping sites where food was always provided ad libitum, or the alternate sites with no food, that as such did not affect *ψ*. The MSORD model also provides two derived (i.e., not included in the likelihood formulation) parameters: (vi) population size (*N*_t_), i.e., the number of individuals occupying the study area in state s in that primary occasion *t*; and (vii) residence time (*R*_t_), which expresses the average number of secondary occasions that an individual spent in the study area for that state s in that primary occasion *t* (Boys et al. 2019; Chabanne et al. 2017).

We modelled demographic parameters in dependence on temporal, spatial, state, and individual covariates that were biologically meaningful to address our working hypotheses. We modelled survival (*S*) and persistence probability (φ) as varying in dependence on primary occasions (‘session’, only for φ) and successive seasonal periods (‘season’) to detect the temporal pattern (H2.1); supplemental food availability (‘feeding state’ in Norway and ‘feeding site’ in Italy) to identify the food effect (H1); sex (‘sex’) to detect the effect of individual traits (H2.2), and ultimately species (only in Italy, H2.3). We considered the probabilities of capture (*p*) and arrival (*pent*) to be dependent on temporal (primary occasions i.e., ‘session’ for *p*, secondary occasions i.e., ‘time’ for *pent*) and seasonal (‘seasonal periods’) variations, supplemental food availability (‘feeding state’‐Norway and ‘feeding site’‐Italy) and species (only in Italy). We modelled *ψ* in dependence on transition of feeding state for Norway and kept it constant for Italy (expressing the probability of transition of animal observability in the trapping grid; see above) (see also Appendix S4 and Table S4 for description of covariates and models building). We fitted the models with the *RMark* package (Laake 2013) in the R program (R Core Team 2023) and used the AICc (corrected Akaike's Information Criterion) for model selection (Burnham and Anderson 2002) with a threshold of ∆AICc ≤ 4. Two models for Norway and three models for Italy were selected as equally parsimonious; among these, we chose for the prediction of parameters the models which retained the covariates that allowed us to better test our hypotheses (Appendix S5).

For both study systems, we applied regression models (Turchin and Ostfeld 1997; Klemola et al. 2002; Huitu et al. 2004) to assess intra‐specific density dependence in the growth rate within the same species, both at sites with and without supplemental food (Appendix S6). These models are also used to test for inter‐specific competition (H2.3) by modelling the growth rate of one species as the response variable and the population density of possible competitor species as the explanatory variable. Specifically, we calculated the species growth rate (*r*_*t*_) between two primary occasions, given the population size at occasion *t* (*N*_*t*_) and *t* + 1 (*N*_*t+*1_). We then modelled r_t_ of each species depending on (i) the ln‐transformed *N*_*t*_ of the same species (intra‐specific density dependence) and (ii) the population size of the other sympatric species (inter‐specific density dependence), both at sites with and without supplemental food. We fitted the models in the R program (R Core Team 2023) using the *modelbased* (Makowski 2020), *tidyverse* (Wickham 2017) and *Sjplot* (Lüdecke 2020) packages.

## 3 Results

### 3.1 Assemblage Description

In Norway, 917 bank voles ( *C. glareolus* ) were captured, accounting for 3976 total capture events. More individuals were captured when supplemental feeding was available (*n* = 462 for 2880 night traps) than when it was not (*n* = 455 for 10,504 night traps). The sex ratio was similar with and without supplemental food availability (Table S3.1, Appendix S3).

In Italy, we discarded data from 22 animals in total, either because they belonged to non‐target species (shrews; one bycatch dormouse) or because they lost the PIT tag (but were identified by the signs of biopsy). Beyond these, 507 animals were captured (Figure S3.2, Appendix S3) belonging to three species: 390 yellow‐necked mice ( *A. flavicollis* ), 8 wood mice ( *A. sylvaticus* ; considered *ensemble* to *A. flavicollis* as *Apodemus* spp.) and 109 bank voles ( *C. glareolus* ), accounting for 1379 capture events in total (926 for *Apodemus* spp. and 453 for *C. glareolus* ). Given the low number of captures of wood mice ( *A. sylvaticus* ) and the intent to discriminate between Murids (i.e., mice and rats) and Arvicoline rodents (i.e., voles, lemmings and muskrats), individuals of yellow‐necked mice ( *A. flavicollis* ) and *A. sylvaticus* were treated together as *Apodemus* spp. We captured more mice where supplemental food was available (*n* = 234 for 21,888 night traps) than in grids where it was not (*n* = 164 for 21,888 night traps), and the opposite for voles (29 and 80, respectively). The sex ratio was slightly different with and without supplemental food, especially in Italy (see Table S3.1, Appendix S3).

### 3.2 Population Demography

#### 3.2.1 Probability of Survival

**FIGURE 3:**
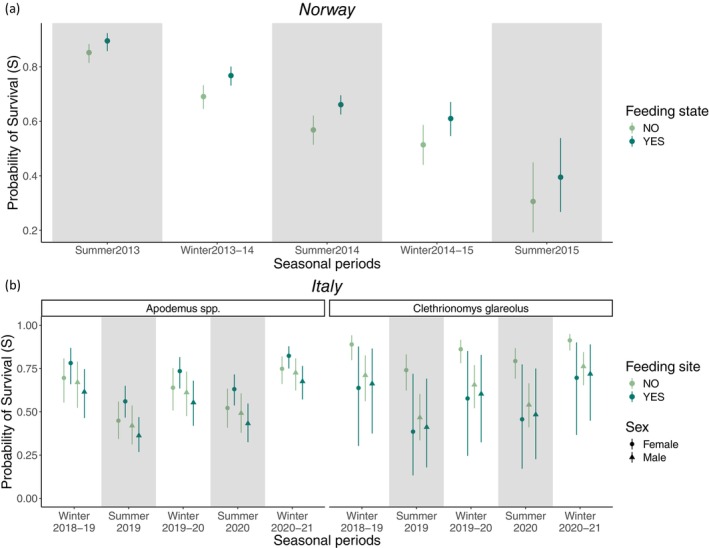
Estimates of survival (*S*) for the detected species in Norway (a) and Italy (b) across seasonal periods, sex (female = circular shape; male = triangular shape) and under different food availability conditions (supplemental food available = dark green; no supplemental food available = light green; ‘Feeding state’ for Norway and ‘Feeding site’ for Italy). Grey shaded bars indicate the summer periods, while white bars denote the winter periods. The comparison between survival estimates in a given winter (overwintering animals) and in the following summer (reproductive season) was not applicable for winter 2012–2013 versus summer 2013 in Norway, and winter 2020–2021 versus summer 2021 in Italy.

For Norway, *S* depended on the additive effect of seasonal periods (H2.1) and supplemental food availability (H1) (Table S5.1). In particular, *S* was higher during winter with respect to the following summer (for those years where data for both seasonal periods were available). Moreover, S was higher when supplemental food was provided than when this was not the case, regardless of the season (Appendix S7, Table S7.1; Figure 3a; for *φ*, *pent* and *p*, see Appendix S8 and Figures S8.1–S8.3, respectively).

In Italy, *S* depended on the seasonal periods (H2.1) and on the interaction between species (H2.3), sex (H2.2) and feeding site (H1) (Table S5.2). In particular, both *C. glareolus* and *Apodemus* spp. showed a similar seasonal pattern, with *S* increasing in winter and declining in summer. At sites without food supplementation, *S* seemed to remain higher in *C. glareolus* than in *Apodemus* spp., but only in females. Indeed, while in *Apodemus* spp. females and males retained a similar survival, in *C. glareolus the* females outperformed males, showing a higher survival. At sites where supplemental food was provided, *S* increased in *Apodemus* spp. females, while slightly decreased in males. Instead, survival in both sexes strongly decreased in *C. glareolus* with respect to what was observed at sites without food supplementation (Appendix S7, Table S7.3; Figure 3b; for *φ*, *pent* and *p*, see Appendix S8 and Figures S8.1–S8.3 respectively; Appendix S9, Figure S9).

#### 3.2.2 Population Size

**FIGURE 4:**
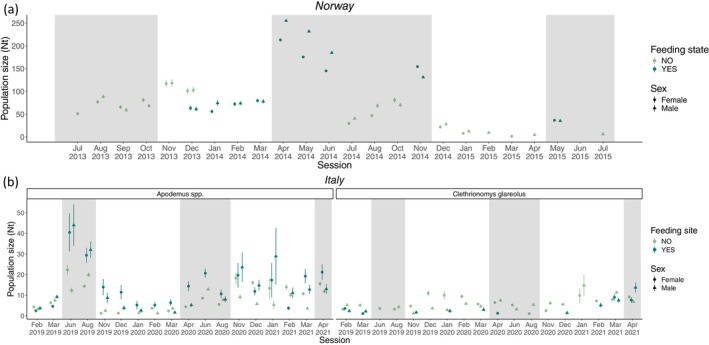
Population size (*N*_*t*_) for the detected species in Norway, 2013–2015 (a) and Italy, 2019–2021 (b) across sessions, sex (female = circular shape; male = triangular shape), and under different food availability conditions (supplemental food available = dark green; no supplemental food available = light green; ‘Feeding state’ for Norway and ‘Feeding site’ for Italy). Grey shaded bars indicate the summer periods, while white bars denote the winter periods. The missing values of *C. glareolus* in the time series (Norway: Feb 2014 and May 2015; Italy: June 2019, August 2019, December 2019, February 2020, June 2020, August 2020, November 2020 and January 2021) are due to lack of estimates from the model in those sessions.

In Norway (Appendix S7, Table S7.2; Figure 4a), when supplemental food was not provided, *C. glareolus* population size was slightly higher in summer than in winter (H2.1) and generally decreased throughout the study period. In the months with availability of supplemental food, the population size was significantly higher (H1) (ANOVA, *p*‐value < 0.0001) for both sexes (H2.2), but the temporal irregularity of sampling and therefore estimates (Appendices S2 and S7) did not allow for inferring any seasonal pattern.

In Italy (Appendix 7, Table S7.4; Figure 4b), when supplemental food was not provided, the population size of *Apodemus* spp. and *C. glareolus* was asynchronous, with peaks in different periods (H2.1), although with no specific pattern shown by sexes (H2.2). In particular, *Apodemus* spp. showed a summer peak followed by a sharp decrease through winter 2019–2020, while in 2020 the population continued to increase after the summer season. Conversely, *C. glareolus* increased during winter and decreased in summer. With supplemental food (H1), *Apodemus* spp. exhibited the same annual pattern but with a higher population size exemplified in both sexes (ANOVA, *p*‐value = 0.01); on the contrary, *C. glareolus* was almost absent where food was provided with no evident differences among sexes, with the exception of a slight recovery only during the last winter sessions in 2020–2021 (ANOVA, *p*‐value = 0.13) (H1, H2.3).

The regression models to assess the patterns of intra‐ and inter‐specific density dependence in *C. glareolus* and *Apodemus* spp. did not show any significant relationship regarding the inter‐specific competition in Italy, neither in the presence nor in the absence of food supplementation (see Appendix S6 and Tables S6.1, S6.2 for further details). Instead, intra‐specific models supported a moderate inverse density dependence in *C. glareolus* in both study systems, regardless of food supplementation (with food *p*‐value < 0.05 in Italy and Norway, with and without food; Appendix S6).

## 4 Discussion

Our descriptive comparison based on field experiments of food manipulation indicates that food availability affects woodland rodent demography along the cyclic‐eruptive continuum of population dynamics, yet is modulated by site‐specific factors such as intra‐and inter‐specific relationships and seasonality, as well as intrinsic individual traits, such as sex. Specifically, survival may be enhanced by food supplementation in voles in Norway and in females' *Apodemus* spp., but not in voles that showed a decrease in survival in both sexes in Italy, where the presence of sympatric wood mice may have modulated the process. Here, the *Apodemus* spp. population may have increased its size throughout the year at sites where food was provided, while the *C. glareolus* population contextually decreased. In the absence of food manipulation, the *C. glareolus* population did not decrease but responded to seasonal fluctuations with an opposite pattern with respect to the sympatric mice. This comparison thus confirms the ecological complexity governing rodent demography, which is shaped by a combination of extrinsic and intrinsic factors varying along latitudinal gradients (see e.g., Lambin et al. (2006) for a similar conclusion for the predation hypothesis).

Well‐known patterns of seasonal demography for rodents living in temperate and boreal climates, that is an alternation between breeding (late spring to early autumn) and non‐breeding (late autumn to early spring) seasons (Eccard and Herde 2013), were clearly observed in our results. This suggests that intrinsic mechanisms, such as the changes of adaptive responses in energy allocation and expenditure during demanding seasons (i.e., maintenance metabolism, behavioural variability, thermoregulation and other metabolic activities), which differed also among sexes, represent the leading forces driving survival, which was higher in winter than in summer, and population size, which conversely was higher in summer than in winter (*supporting* H2.2). Our survival estimates refer to apparent survival, which is the product of true survival and the probability that an individual does not permanently emigrate from the site (i.e., fidelity/dispersal) (Sandercock 2006). These estimates may be negatively biased by non‐random temporary emigration (Schaub et al. 2004), unless it is explicitly included in the CMR models. As we accounted for random emigration only in the transience across states, we interpret our results in light also of the dispersal seasonal‐dependent movements that may have occurred, together with habitat quality intended as climate, vegetation structure and type, and predation that in turn may shape rodent demography. Specifically, favourable environmental conditions, including mild climatic conditions and food quality, stimulate reproduction (Steinlechner and Puchalski 2003) from spring till autumn, with several generations of new‐borns that increase the population size. During this period, the survival is lowered (*supporting* H2.1) by a combination of (i) high emigration rate of overwintered individuals (Olenev and Grigorkina 2014); (ii) deaths of early‐born and immature animals that increased their competitiveness for finding food and mating (Flowerdew et al. 1985) and (iii) a strong predation pressure by avian and mammalian predators that contributes to higher mortality in early‐born animals (Haapakoski et al. 2012). In winter, lower carrying capacity, resulting from limited food replenishment and harsh climate conditions, cannot support the summer population size, leading to a decline in winter abundance (*supporting* H2.1). In this period, immature individuals' are prone to disperse, while late‐born animals (i.e., overwintering individuals) tend to increase their survival chances due to shy and careful personalities (Eccard and Herde 2013), decreasing their predation risk and guaranteeing the bulk for the following year's spring population. It is noteworthy that the observed variations in survival across sexes in Italy among the two examined rodent species (*supporting* H2.2) may be indicative of the site fidelity expressed by apparent survival and therefore of rodent social and spatial behaviours. In *Apodemus* spp. in sites without supplemental food (i.e., with sparse and patchily distributed natural resources, such as seeds), females and males may increase their travel distance with a greater overlap in their home ranges (Stradiotto et al. 2009; Puig‐Gironès and Pons 2023). This results in a uniform distribution of sexes in the area, with no evident effects on apparent survival. Conversely, in *C. glareolus* , whose diet relies on abundant and evenly distributed resources, such as grasses and sedges, females become more spatially clumped (Ostfeld 1990), avoiding emigration and increasing survival estimates of apparent survival thereafter.

Our latitudinal comparison highlights that the effect of food provisioning on *C. glareolus* survival was more marked at northern latitudes (*partially supporting* H1; *supporting* H2.1), likely due to prolonged and harsher seasons, scarcity of food resources, and delayed phenology (Korslund and Steen 2006; Haapakoski and Ylönen 2013). Here, food does not substantially alter rodent population cycles but interacts with other factors as predation by affecting rodent ecology (e.g., density, movement, survival) (Oli 2019). Indeed, at those latitudes, harsh conditions increase the energetic costs for thermoregulation and reproduction (Ylönen and Eccard 2004), emphasising the importance of food availability in modulating survival (Rémy et al. 2013; Johnsen et al. 2017). Conversely, in Italy, *C. glareolus* experienced a decrease in survival with food supplementation in both sexes (*not supporting* H1). Based on Rémy et al. (2013), we would have expected higher survival where food was provided, especially in females that usually aggregate with concentrated food. Instead, the decline in survival of *C. glareolus* may be a consequence of inter‐specific competition that may have arisen between *C. glareolus* and *Apodemus* spp. (*supporting* H2.3). As concentrated food resources enhanced *Apodemus* spp. abundance, this may have depleted other resources beyond those provided at feeding sites, further depriving *C. glareolus* and reducing its survival for both sexes. Alternatively, the reduction in vole survival in Italy may be an effect of the different types of food offered in the two study systems (i.e., oats in Norway and corn in Italy), which could have biased survival estimates due to food habituation. For instance, although being an attractive bait, corn is considered a low‐quality food, reducing the energetic benefits (De Carvalho Braga et al. 2015) and being thus less selected by voles. Furthermore, the decline in survival reported for *C. glareolus* may be related to the environmental context that characterises the two study systems. In particular, although the study systems are characterised by homogeneous coniferous forest ecosystems and we accounted for the seasonal variability, intrinsic microhabitat features (i.e., understorey, vegetation structure and type) and different predator communities may have altered the demographic responses of voles (Dorigo et al. 2021; Puig‐Gironès and Pons 2023; Brehm and Mortelliti 2024) to food supplementation. At the same time, however, food supplementation in Italy has improved female survival in *Apodemus* spp. (*supporting* H1 and H2.2). In particular, food resources may have promoted the survival of granivorous *Apodemus* spp. on the one hand, confirming other findings (Flowerdew and Gardner 1978; Díaz and Alonso 2003); while on the other hand, promoted aggregation of females around the food source (Rémy et al. 2013), thus improving apparent survival of *Apodemus* spp.

The latitudinal comparison has also shown that the availability of trophic resources is the major driver of population size. In general, when resources are abundant, immigration is favoured, home ranges shrink (see e.g., Taitt and Krebs 1981; Stradiotto et al. 2009), and population size increases thereafter (Le Galliard et al. 2012; Rémy et al. 2013). This is also supported by our MSORD models, in which individual residence time, which prompts familiarity with the distribution of resources in a defined area (a proxy for home range; Piper 2011), increased in sites with food supplementation. Interestingly, *C. glareolus* showed an overall decrease in population size trend in Norway (*partially supporting* H1), due to the upcoming crash phase of 4‐year periodic cycles (as detected in Johnsen et al. 2019), but population size attained higher levels when food was provided, further underlying the importance of food resources for the demographic patterns of rodents at northern latitudes (*partially supporting* H1 and H2.1). In Italy instead, population abundance of both *Apodemus* spp. and *C. glareolus* kept increasing from summer 2020 to winter 2020–2021, following a beech, oak and spruce mast event (evidenced in the pollen quantities reported in Marini, Tagliapietra, et al. 2023), which provided overabundant spatially dispersed food resources (*supporting* H1 and H2.1). This may have minimised any confounding effect emerged from artificial concentrated food supply on rodent demography, confirming the crucial role of spatial distribution of food resources (Rémy et al. 2013). In this context, food supplementation affected the two species differently, possibly due to the inter‐specific interactions (*supporting* H2.3), favouring the less generalist but dominant *Apodemus* spp. The decrease of *C. glareolus* population size in the presence of supplemental food may also be due to other external factors governing rodent demography in natural settings. Indeed, at southern latitudes, milder conditions in forestry environments promoted the co‐occurrence of multiple species, in accordance with latitudinal biodiversity gradients (Araújo and Costa‐Pereira 2013; Mannion et al. 2014). The sympatry of *C. glareolus* and *Apodemus* spp. observed in Italian woodlands emerged with an asynchronous seasonal pattern of their population size, that is opposite peak and crush seasonal phases between species, that we interpreted as evidence of temporal niche partitioning performed by *C. glareolus* to avoid the dominant *Apodemus* spp. (Amori et al. 2015; Casula et al. 2019; Viviano et al. 2022). However, the excess of food availability in our experiment may have altered this process. In particular, we speculate that chronic overabundance of long‐term concentrated food resources might have triggered competition phenomena between *Apodemus* spp. and *C. glareolus* , emerging for instance as interference, exploitative, or apparent competition. This might have led to the collapse of temporal niche partitioning and consequent *C. glareolus* displacement and reduction of survival rates, which is what we actually observed at sites with food supplementation (*supporting* H2.3). When food is confined, it becomes a defensible resource for species that cache food in larger hoards such as *Apodemus* spp. (Zwolak, Bogdziewicz, and Rychlik 2016). This potentially leads to aggressive interactions (interference competition), eventually causing the collapse of *C. glareolus* (see e.g., Eccard and Ylönen 2007). Moreover, *Apodemus* spp. might have depleted other resources beyond those provided at feeding sites, further depriving *C. glareolus* (exploitative competition) (Gilad 2008). Furthermore, rodent predators, such as birds of prey and foxes, may have benefited from the increase of *Apodemus* spp. populations, requiring more prey and, thus, eventually negatively affecting *C. glareolus* through increased predation pressure (apparent competition) (Holt 1977). In nature, these competitive mechanisms often co‐occur, and their effects are difficult to separate (Schmidt et al. 2005; Sinclair and Krebs 2002). Although our results might suggest the existence of some form of inter‐specific competition, we acknowledge that we did not find any direct evidence of this, as emerged from the lack of significance in the inter‐specific density dependence models. This lack of evidence may be imputed to a delayed effect of the long‐term historical food manipulation. It is also plausible that this is due to a lack of power of our sample size, because of the extremely low numbers of *C. glareolus* when *Apodemus* spp. was abundant. However, if on one hand we did not statistically confirm a direct relationship between sympatric species; on the other hand, we observed inverse intra‐specific density dependence, namely Allee effect, in voles in both study systems, being particularly evident at sites provided with food. Under these conditions, per capita growth rate is reduced at low density, resulting in destabilised populations. In both study systems, populations of voles reached relatively low values of population size. In Norway, the Allee effect may contribute importantly to population lows and crashes in cyclic multiannual rodent dynamics (Jankovic and Petrovskii 2014). In Italy, the same effect may indicate the presence of some kind of resource exploitation driven by inter‐specific competition, which rebounds on intra‐specific relationships (Etienne et al. 2002). This can be confirmed also with the absence of Allee effect in *Apodemus* spp. populations, in which aggregation of individuals due to food supplementation enabled high density, reducing Allee effect threshold (Jorge and Martinez‐Garcia 2024). Certainly, the combination of Allee effects and competition needs further experimental work to be clarified.

It is worth noting that the experimental designs in the Norwegian and Italian systems differed in some details (i.e., variability in food distribution, food type, grid pattern, timing of trapping session), which, however, we believe do not affect their comparability and, therefore, the validity of our findings. The two study systems are located at the extremes of the boreal–temperate latitudinal gradient and are characterised by differences in ecological interactions and species‐specific adaptations. Therefore, we qualitatively compared the two similar but independent study systems by (i) accounting for environmental conditions, seasonal variation, intrinsic individual traits, and finally, where possible, inter‐specific interactions; (ii) interpreting the results within the same theoretical framework while adapting the quantitative approach to each experimental design. In this way, we were able to assess the effect of food on rodent demography in two contrasting population dynamics systems by explicitly testing its role in natural settings, accounting for the confounding factors (i.e., seasons, climate, sex, inter‐specific competition) that may interact in shaping this relationship while considering methodological differences. In particular, the temporal variation in food availability in Norway has the potential to elicit a delayed response in demographic parameters, such as survival and reproduction, following food manipulation. This response exhibits a lag time, akin to the delayed effects observed in rodents during mass seeding production or primary productivity (Lobo and Millar 2013; Rossi and Leiner 2024), that has emerged in Italy when a mast event occurred. Despite the differences in experimental design, including the irregular temporal variation of moderate food distribution in Norway and the spatial variation with abundant food in Italy, in Norway we cannot observe the delayed effect of food on demography due to the moderate supplementation and the short sampling period, overlapping also partially with the crash phase of the population cycle. Although it was not possible to implement a time‐lagged variable in the analysis, we considered the spatio‐temporal differences by applying transition probability across feeding states in the Norwegian models. At the same time, when analogous temporal fluctuations in food availability were observed in Italy, for example, during the mast event, an initial delayed effect on population size was detected due to the abundant food resources available, thus confirming the congruence between the two study systems. Undoubtedly, further long‐term experimental research is necessary to elucidate the delayed demographic responses to varying food availability in these contexts.

Furthermore, despite their different shape and size, both grid patterns (cross‐shaped 16‐traps grid in Norway and squared 64‐traps grid in Italy) account for the “edge‐effect” and are both suitable for demographic and density studies, being thus comparable (McCleery et al. 2021). Also, differences in timing in trap check (12 h in Norway and 24 h in Italy) would not strongly influence estimation in demographic parameters, as the trapping nights, on which trapping effort is based, are the same across systems, that is 3 nights (McCleery et al. 2021). The evening trap check performed in Norway was carried out mainly to prevent individual mortality during night due to harsh climate. Moreover, differences in the type of supplemental food (a mixture of oat and sunflower seeds in Norway; corn in Italy) do not affect their attractiveness for our target species, being appropriate for both granivorous species such as *Apodemus* spp., and also cricetids such as *Clethrionomys* species that hold an intermediate position between granivores and typical herbivores (see Butet and Delettre 2011; Gasperini et al. 2018). Finally, spatial distribution of supplemental food also changed across study systems (spatially distributed in Norway; spatially centralised in Italy). Spatial distribution of food may have influenced individual spatial behaviour and movements, although not across trapping grids, enhancing population growth, as described in enclosures by Rémy et al. (2013).

## 5 Conclusions

Overall, despite the evident heterogeneity between the two study systems, the qualitative comparison interpreted within the same theoretical framework employing the same quantitative approach and accommodating the different designs allowed the unravelling of both similarities and dissimilarities in the demographic responses of mast‐driven populations of Italian rodents and Norwegian cyclic populations. According to Lambin et al. (2006), although it is conceivable to find the proximate factors driving oscillations of population dynamics in different regions, it is also crucial to investigate the explanations for the common patterns that we can find in different rodent populations. This is especially prompted for those species, such as*Clethrionomys*and*Microtus*species, that have regular cyclic dynamics in some parts of their distribution and irregular outbreak dynamics in other parts, responding differently due to their life‐history strategies, ecological niches, and local adaptations. In this sense, we confirmed that food availability represents a key driver of rodent demography influencing rodent population size and survival rates in different systems, albeit modulated also by seasonal fluctuations and intrinsic traits. However, rodent population dynamics can be influenced also by predation, competition, habitat quality beyond food resources, and diseases. In this sense, we advocate for a multi‐faceted approach that simultaneously evaluates changes in rodent demography across different population dynamics by considering the interplay of intrinsic and extrinsic factors, such as density dependence and habitat suitability, as well as the overlapping of sympatric, competitive, or predatory species, and pathogen susceptibility.

In perspective, these studies are especially relevant when considering climate change. Specifically, the compensatory shifts in demographic parameters, such as survival rates and population size observed in Italy at feeding sites, may reflect changes expected in northern latitudes due to climate change. In particular, as environmental conditions become more permissive in the boreal‐temperate zone, cold climate‐constrained species may expand northward or at higher altitudes (Moritz et al. 2008; Sokolova et al. 2024). Moreover, more frequent mast events towards northern latitudes, predicted under climate change, may increase populations of granivorous species, intensifying seed predation (i.e., weak predator satiation, see Zwolak et al. 2022), while reducing viable seed production (Bogdziewicz et al. 2021). Both these possible scenarios could be mitigated or limited by some ecological and demographic mechanisms (i.e., density dependence, local extinctions, habitat suitability) but could also result in spatial (by sharing the same space within the geographical area or range) and niche (by sharing the same roles or resource use within the environment) overlap with other cold‐adapted species or encroachment with species that have evolved to thrive in warmer conditions (Markova et al. 2018). This overlap can lead to different outcomes in terms of resource availability, altering direct and indirect inter‐specific dynamics within the rodent community, with some species thriving while others face increased risk of extinction or niche displacement.

## Author Contributions

**Giulia Ferrari:** conceptualization (equal), data curation (equal), formal analysis (equal), investigation (equal), methodology (equal), writing – original draft (equal), writing – review and editing (equal). **Olivier Devineau:** conceptualization (equal), data curation (equal), formal analysis (equal), methodology (equal), resources (equal), supervision (equal), writing – review and editing (equal). **Valentina Tagliapietra:** conceptualization (equal), investigation (equal), methodology (equal), resources (equal), supervision (equal), writing – review and editing (equal). **Kaja Johnsen:** data curation (equal), investigation (equal), methodology (equal), writing – review and editing (equal). **Federico Ossi:** methodology (equal), writing – review and editing (equal). **Francesca Cagnacci:** conceptualization (equal), methodology (equal), resources (equal), supervision (equal), writing – review and editing (equal).

## Ethics Statement

The fieldwork protocol was approved by the Norwegian Animal Research Authority, i.e., Norwegian Food Safety Authority (FOTS 15309, 15585), in Norway and by the Provincial Wildlife Management Committee of the Autonomous Province of Trento, in Italy (Authorization n. 663; date: 23/04/2015).

## Conflicts of Interest

The authors declare no conflicts of interest.

## Supporting information


Appendix S1.

## Data Availability

The authors have archived data and code used for analyses reported in this manuscript on Zenodo and registered a DOI publicly accessible (https://doi.org/10.5281/zenodo.13349536).
