# Supplementary material for: Effect of Resource Abundance on Woodland Rodents' Demography at Latitudinal Extremes in Europe
Source: Ecol Evol. 2025 Jun 2;15(6):e71466. doi: 10.1002/ece3.71466 (PMC12129823; doi:10.1002/ece3.71466)
Supplement: Supplementary file 1 — Appendix S1. [file ECE3-15-e71466-s001.docx]

**Supporting Information**

**Appendix S1: Trapping grid design**

Trapping grid design. The panel A shows the cross-shaped design with 16 traps used in Norway, while in the panel B the square design of 64 traps applied in Italy is represented.

**
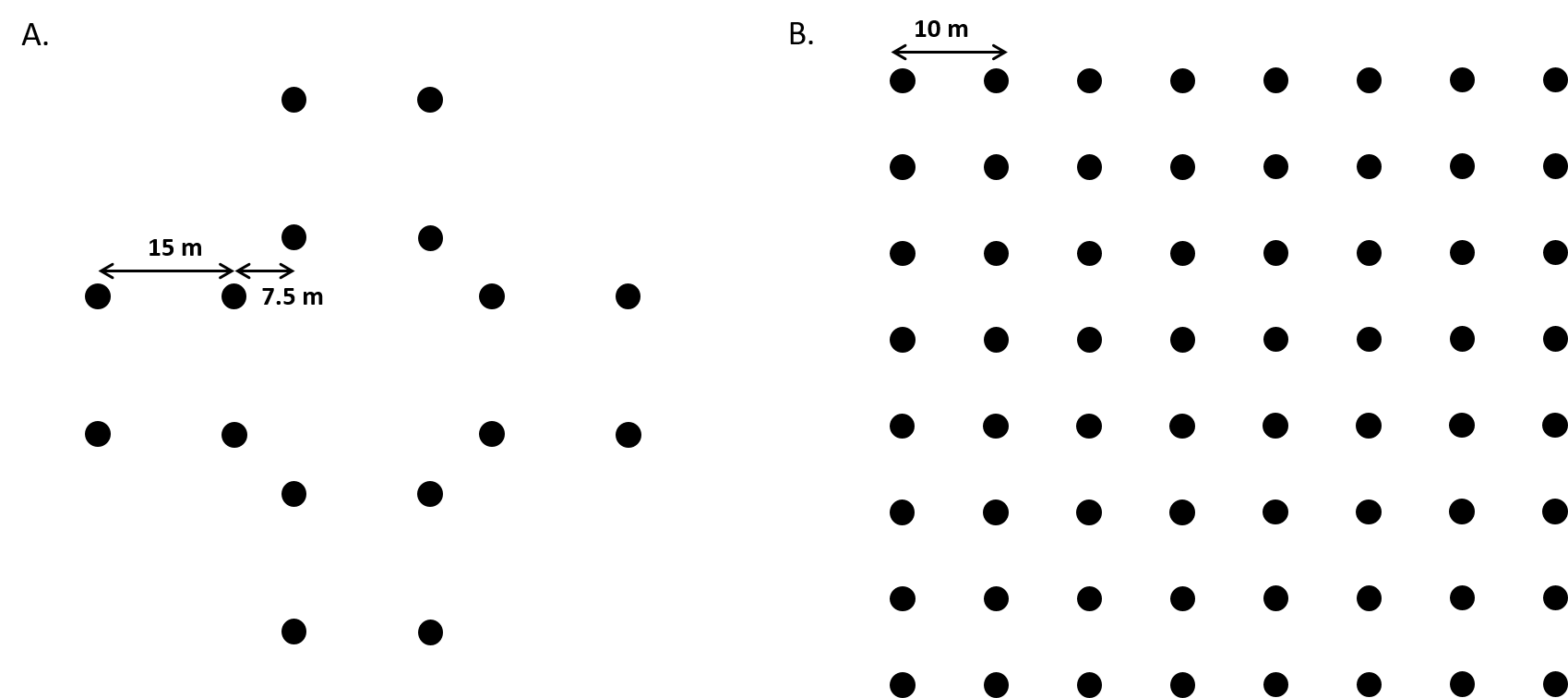
**

**Appendix S2: Trapping sessions**

In Norway the dataset included 26 primary occasions with two to seven secondary occasions from 2013 to 2015 (Table S2.1), while Italian trapping data consisted of 18 primary occasions with 3 secondary occasions each from 2019 to 2021 (see Table S2.2). Since the analysis used calendar months as temporal categorical variable, we decided to unify the secondary occasions of captures occurring in different sessions but within the same month (e.g. March 2019 in Italy and July 2013 in Norway).

**Table S2:** Primary and secondary capture occasions in Norway (**Table S2.1**) and Italy (**Table S2.2**). Grey-shaded occasions in Norway showed when food was provided at trapping grids (see main text).

| Seasonal periods | Summer 2013 | | | | | | | | Winter 2013/2014 | | | | |
| --- | --- | --- | --- | --- | --- | --- | --- | --- | --- | --- | --- | --- | --- |
| Primary occasion | 1 | | | | | 2 | 3 | 4 | 5 | 6 | 7 | 8 | 9 |
| Secondary occ 1 | 03 Jul | | | 22 Jul | | 10 Aug | 09 Sep | 09 Oct | 09 Nov | 09 Dec | 20 Jan | 17 Feb | 17 Mar |
| Secondary occ 2 | 04 Jul | | | 23 Jul | | 14 Aug | 10 Sep | 10 Oct | 11 Nov | 10 Dec | 21 Jan | 18 Feb | 18 Mar |
| Secondary occ 3 | 05 Jul | | | 24 Jul | | 15 Aug |  |  | 12 Nov | 11 Dec |  | 19 Feb | 19 Mar |
| Secondary occ 4 | 10 Jul | | |  | | 16 Aug |  |  | 13 Nov | 12 Dec |  | 20 Feb | 20 Mar |
| Secondary occ 5 |  | | |  | | 17 Aug |  |  | 14 Nov |  |  |  |  |
|  | Summer 2014 | | | | | | | | Winter 2014/2015 | | | | |
| Primary occasion | 10 | | 11 | | 12 | 13 | 14 | 16 | 17 | 18 | 19 | 20 | 21 |
| Secondary occ 1 | 22 Apr | | 19 May | | 23 Jun | 21 Jul | 18 Aug | 13 Oct | 03 Nov | 08 Dec | 12 Jan | 10 Feb | 18 Mar |
| Secondary occ 2 | 23 Apr | | 20 May | | 24 Jun | 22 Jul | 19 Aug | 14 Oct | 04 Nov | 09 Dec | 13 Jan | 11 Feb | 19 Mar |
| Secondary occ 3 | 24 Apr | | 21 May | | 25 Jun | 23 Jul | 20 Aug | 15 Oct | 05 Nov | 10 Dec | 14 Jan | 12 Feb | 20 Mar |
| Secondary occ 4 | 25 Apr | | 22 May | | 26 Jun | 24 Jul | 21 Aug | 16 Oct | 06 Nov | 11 Dec | 15 Jan | 13 Feb |  |
|  | Summer 2015 | | | | | | |  |  |  |  |  |  |
| Primary occasion | 22 | 23 | | | 24 | 25 | 26 |  |  |  |  |  |  |
| Secondary occ 1 | 14 Apr | 18 May | | | 16 Jun | 13 Jul | 11 Aug |  |  |  |  |  |  |
| Secondary occ 2 | 15 Apr | 19 May | | | 17 Jun | 14 Jul | 12 Aug |  |  |  |  |  |  |
| Secondary occ 3 | 16 Apr | 20 May | | | 18 Jun | 15 Jul | 13 Aug |  |  |  |  |  |  |
| Secondary occ 4 |  | 21 May | | | 19 Jun | 16 Jul | 14 Aug |  |  |  |  |  |  |

**Table S2.1**

| Seasonal periods |  |  | | Winter 2018/2019 | | | Summer 2019 | | |  |
| --- | --- | --- | --- | --- | --- | --- | --- | --- | --- | --- |
| Primary occasion |  | |  | 1 | 2 | | 3 | | 4 |  |
| Secondary occ 1 |  | |  | 19 Feb | 05 Mar | 19 Mar | | 25 Jun | 27 Aug |  |
| Secondary occ 2 |  | |  | 20 Feb | 06 Mar | 20 Mar | | 26 Jun | 28 Aug |  |
| Secondary occ 3 |  | |  | 21 Feb | 07 Mar | 21 Mar | | 27 Jun | 29 Aug |  |
|  | Winter 2019/2020 | | | | | | | Summer 2020 | | |
| Primary occasion | 5 | | 6 | 7 | 8 | 9 | | 10 | 11 | 12 |
| Secondary occ 1 | 12 Nov | | 10 Dec | 14 Jan | 11 Feb | 04 Mar | | 21 Apr | 30 Jun | 25 Aug |
| Secondary occ 2 | 13 Nov | | 11 Dec | 15 Jan | 12 Feb | 05 Mar | | 22 Apr | 01 Jul | 26 Aug |
| Secondary occ 3 | 14 Nov | | 12 Dec | 16 Jan | 13 Feb | 06 Mar | | 23 Apr | 02 Jul | 27 Aug |
|  | Winter 2020/2021 | | | | | | | Summer 2021 | |  |
| Primary occasion | 13 | | 14 | 15 | 16 | 17 | | 18 | |  |
| Secondary occ 1 | 17 Nov | | 15 Dec | 12 Jan | 23 Feb | 9 Mar | | 7 Apr | |  |
| Secondary occ 2 | 18 Nov | | 16 Dec | 13 Jan | 24 Feb | 10 Mar | | 8 Apr | |  |
| Secondary occ 3 | 19 Nov | | 17 Dec | 14 Jan | 25 Feb | 11 Mar | | 9 Apr | |  |

**Table S2.2**

**Appendix S3: Preliminary exploratory analyses**

**Norway – Evenstad site**

In Norway, only one species was captured (*Clethrionomys glareolus*). The density of captures had a similar pattern in females and in males with two annual peaks, during early summer and autumn (Fig. S3.1; Table S3.1), and a general trend towards a decrement of captures across years.


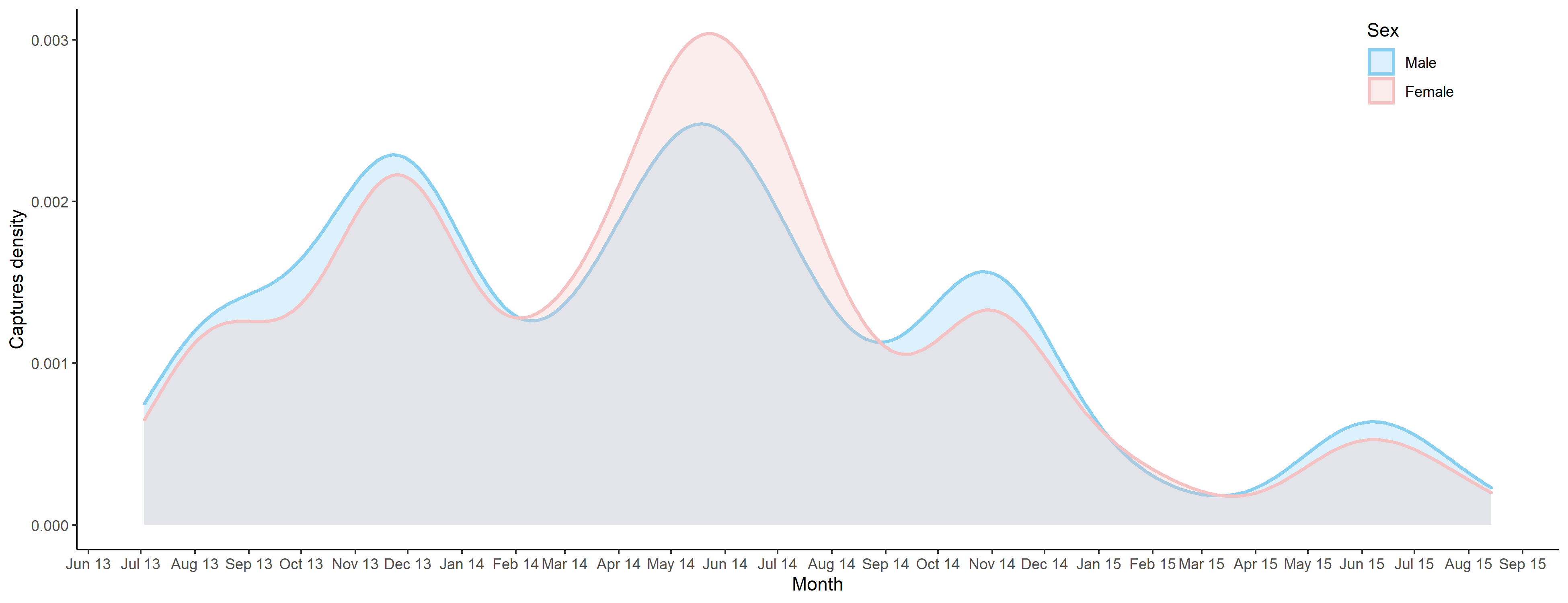


**Figure S3.1**: Monthly capture densities of females (pink) and males (light blue) *C. glareolus* in Norway (2013-2015).

**Italy – Cembra site**

In Italy, three species were detected *(Apodemus sylvaticus, A. flavicollis* and *Clethrionomys glareolus*), and the first two species were grouped as *Apodemus* spp.. When we compared the density of captures across trapping occasions, we detected a cyclic asynchronous pattern between the species (Fig. S3.2). In particular, *Apodemus* spp. were captured more during summer compared to winter, with the exception of winter 2020-21. On the contrary, *C. glareolus* was captured more in winter than in summer.


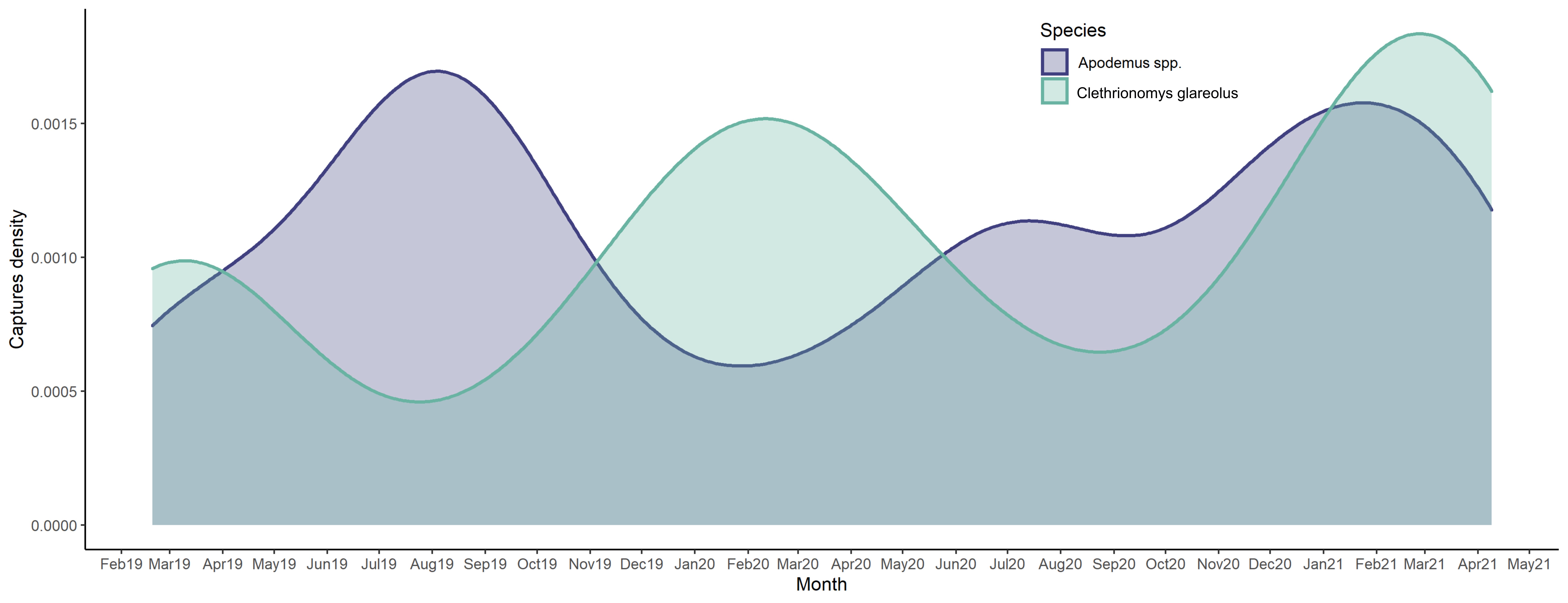


**Figure S3.2**: Monthly capture densities of *Apodemus* spp. (in purple) and *Clethrionomys glareolus* (in green) in Italy (2019-2021).

When breaking down the captures by sex, we found slightly shifted peaks for females and males in the three species, although the proportion of captures were similar (Fig. S3.3; Table S3.1).


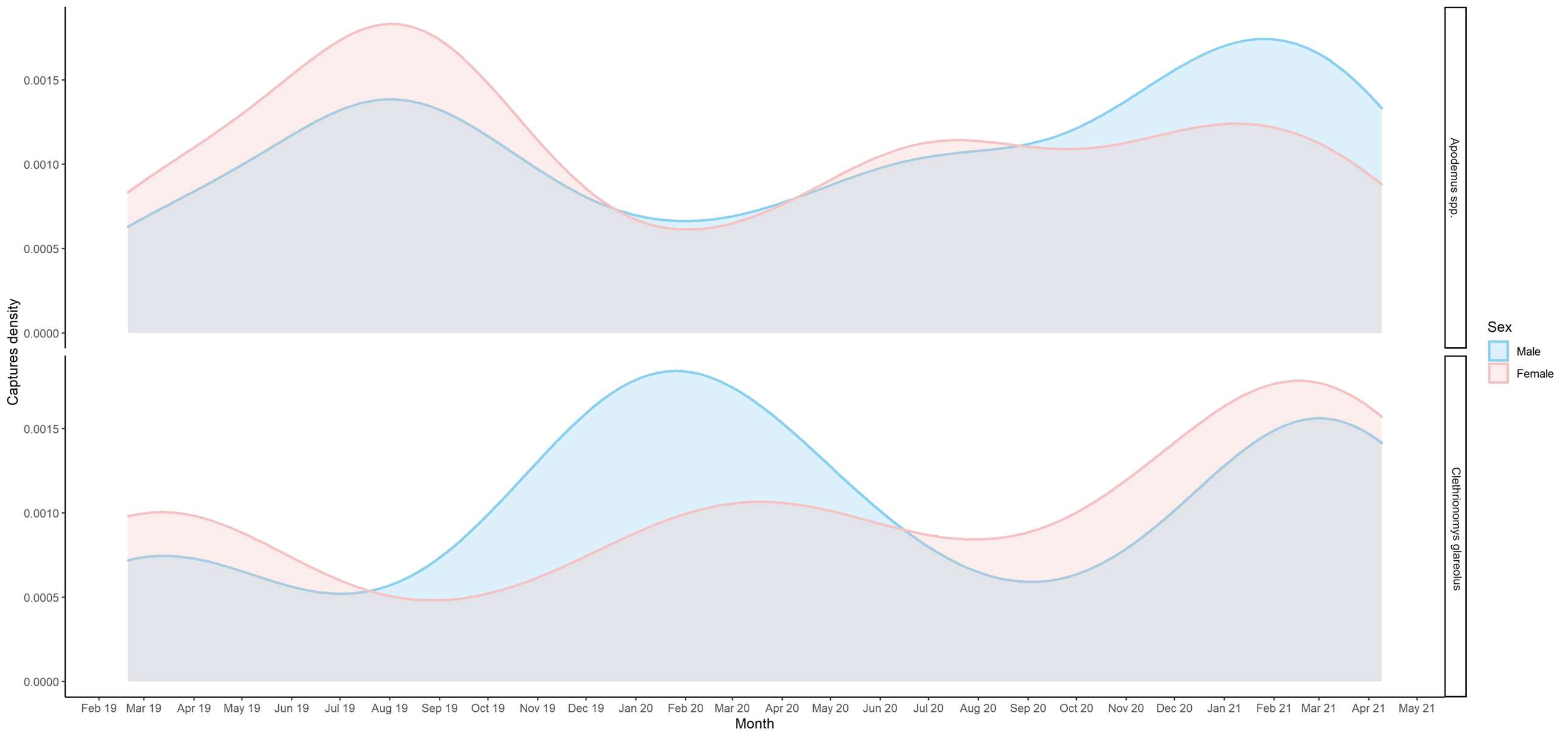


**Figure S3.3**: Monthly capture densities of *Apodemus* spp. and *Clethrionomys glareolus* by sex. Pink area = female; light blue area = males (Italy, 2019-2021).

**Table S3.1**: Summary of the number of captures by sex in presence and absence of supplemental food for *C. glareolus* (Norway and Italy), and *Apodemus* spp. (Italy only).

|  | *Clethrionomys glareolus* | | *Apodemus spp.* | |
| --- | --- | --- | --- | --- |
| Site | Feeding no | Feeding yes | Feeding no | Feeding yes |
| Norway |  |  |  |  |
| *Females* | *212* | *213* |  |  |
| *Males* | *243* | *249* |  |  |
| **Total n. individuals** | **455** | **462** |  |  |
| **Total captures** | **1927** | **2049** |  |  |
| Italy |  |  |  |  |
| *Females* | *34* | *10* | *84* | *117* |
| *Males* | *46* | *19* | *80* | *117* |
| **Total n. individuals** | **80** | **29** | **164** | **234** |
| **Total captures** | **391** | **62** | **440** | **486** |

**Appendix S4: Model building**

We identified a set of potentially biological meaningful covariates to disentangle the effect of supplemental food availability, controlling also for seasonal variation and sympatric species co-occurrence, on rodent demography, and specifically:

(i) temporal covariates:

- **seasonal periods (acting as an ordinary variable)**: successive binned time intervals based on seasonal periods throughout the study duration: winter (from November to March included) and summer (from April to October included);
- **time**: temporal effect within secondary occasions, i.e. daily **secondary trapping occasions;**
- **session**: temporal effect across primary occasions, i.e. monthly **primary trapping occasions**.

(ii) state/spatial covariate:

- **feeding state** of the site (Norway: spatio-temporally defined) and **feeding site** (Italy: spatially defined): presence (treatment) *vs* absence (control) of *ad libitum* supplemental feeding **(binary variables: yes/no)**.

(iii) individual covariate:

- **species (categorical variable;** Italy only**)**: *Apodemus* spp. and *Clethrionomys glareolus*.
- **sex (categorical variable)**: female and male.

In CMR modelling, a main model is described by sub-models estimating each parameter, or response variables, in dependence on several covariates, or explanatory variables (Laake and Rexstad 2008). In particular, multistate open robust design (MSORD) is defined by five parameters (apparent survival (*S*), arrival probability (*pent*), persistence probability (*φ*), capture probability (*p*) and transition probability (*ψ*)).

For modelling each parameter, we chose those covariates that were biologically meaningful in our study systems, while being constrained by a relatively small sample size (i.e., few recaptures). In particular, we modelled apparent survival (*S*) and persistence probability (φ) as varying in dependence on primary occasions (‘session’, only for φ) and successive seasonal periods (‘seasonal periods’) to detect the temporal pattern, supplemental food availability (‘feeding state’ in Norway and ‘feeding site’ in Italy) to identify the food effect, species (only in Italy) to evaluate species co-occurrence, and ‘sex’ to detect intrinsic individual variation. We considered the probabilities of capture (*p*) and arrival (*pent*) to be dependent on temporal variations (primary occasions i.e. ‘session’ for *p*, secondary occasions i.e. ‘time’ for *pent*), temporal periods (‘seasonal periods’), supplemental food availability (‘feeding state’-Norway and ‘feeding site’-Italy), species (only in Italy) and sex (‘sex’ for *p*). We modelled *ψ* in dependence on transition of feeding state for Norway, and kept it constant for Italy (expressing the probability of transition of animal observability in the trapping grid). Table S4 summarizes the list of covariates or combination of covariates used to build the sets of sub-models (univariate, additive, or with two-way interactions among covariates) that were subsequently combined into main models.

Then we used a model selection based on AICc scores to rank the main models composed by sub-models and we retained the models with ∆AICc ≤ 4 as equally plausible (Burnham and Anderson 2002). Among these models, we chose the best model as the more biologically meaningful (Appendix S5).

**Table S4**: Covariates and combination of covariates used to build the set of sub-models for each demographic parameter in Norway (**Table S4.1**) and Italy (**Table S4.2**). Legend: ‘S’ = apparent survival; ‘pent’ = arrival probability; ‘φ’ = persistence probability; ‘p’ = capture probability; ‘ψ’ = transition probability, set as constant in Italy and dependent on feeding states in Norway; ‘session’ = primary trapping occasions; ‘time’ = secondary trapping occasions; ‘feeding site’ = sites with supplemental food (only in Italy); ‘feeding state’ = supplemental food at sites (only in Norway); ‘feeding state : to feeding state’ = transition between feeding states (only in Norway); ‘species’ = rodent species (only for Italy); ‘seasonal periods’ = seasonal variation across years; ‘sex’ = individual sex.

| **Table S4.1** | S | pent | φ | p | Ψ |
| --- | --- | --- | --- | --- | --- |
| feeding state : to feeding state |  |  |  |  | X |
| state : session |  |  |  |  |  |
| feeding state : seasonal periods | X | X | X | X |  |
| feeding state + session |  | X | X | X |  |
| feeding state + seasonal periods | X | X | X | X |  |
| stratum + period + sex | X |  |  | X |  |
| stratum + sex |  |  |  | X |  |
| feeding state | X | X | X | X |  |
| seasonal periods | X | X | X | X |  |
| session | X | X | X | X |  |
| sex | X |  |  |  |  |

| **Table S4.2** | S | pent | φ | p | ψ |
| --- | --- | --- | --- | --- | --- |
| feeding site: seasonal periods : species : sex | X |  |  |  |  |
| feeding site: seasonal periods : species | X |  |  |  |  |
| feeding site: seasonal periods : species + sex | X |  |  |  |  |
| feeding site: seasonal periods : sex + species | X |  |  |  |  |
| feeding site: species : sex + seasonal periods | X |  |  |  |  |
| feeding site : seasonal periods |  | X |  | X |  |
| feeding site : seasonal periods + sex | X |  |  |  |  |
| feeding site : seasonal periods + species | X |  |  |  |  |
| feeding site : species + seasonal periods | X |  |  |  |  |
| feeding site : species + seasonal periods + sex | X |  |  |  |  |
| feeding site + seasonal periods + species + sex | X |  |  |  |  |
| feeding site + seasonal periods + species | X |  |  |  |  |
| feeding site + seasonal periods + sex | X |  |  |  |  |
| feeding site + species + sex | X |  |  |  |  |
| species + seasonal periods + sex | x |  |  |  |  |
| feeding site + session + sex |  |  |  | X |  |
| feeding site + seasonal periods | X | X |  | X |  |
| feeding site + session |  |  |  | X |  |
| feeding site + species | X |  |  |  |  |
| seasonal periods + species | X |  |  |  |  |
| species + sex | X |  |  |  |  |
| feeding site + sex | X |  |  |  |  |
| seasonal periods + sex | X |  |  |  |  |
| feeding site | X | X | X | X |  |
| seasonal periods | X | X | X | X |  |
| session | X |  |  | X |  |
| time |  | X |  |  |  |
| species | X | X | X | X |  |
| 1 | X | X | X | X | X |

**Appendix S5: Model selection**

In Norway, we found one model with ∆AICc ≤ 4 (Table S5.1). We decided to keep the model which had the lowest AIC score, which was also the most biologically meaningful (‘feeding state : session’ effect on *S*, ‘seasonal periods + state’ on *pent*, ‘session’ + ‘feeding state’ on *φ* and ‘feeding state’ on *p*).

In Italy, the model selection returned eight models with ∆AICc ≤ 4 (Table S5.2) Although Model1 was the most parsimonious model with less parameters and lowest AICc, we selected Model3 as best model because it allowed us to test our hypotheses. Specifically, Model 3 retained the interaction ‘feeding site: species : sex’, and the additive effect of the ‘seasonal period’ on *S*; ‘time’ on *pent*; ‘species’ on *φ* and finally, the additive effect of ‘feeding site’ and ‘session’ on *p*.

**Table S5**: Model selection of Norwegian (**Table S5.1**) and Italian (**Table S5.2**) rodent population parameters and capture parameters. The retained models (i.e. ∆AICc<4) are reported, as well as the first model below the AICc threshold (in italic). The model in bold denotes the one that we retained to test our hypotheses. Legend: ‘S’ = apparent survival; ‘pent’ = arrival probability; ‘φ’ = persistence probability; ‘p’ = capture probability; ‘ψ’ = transition probability, set as constant in Italy and dependent on feeding states in Norway; ‘session’ = primary trapping occasions; ‘time’ = secondary trapping occasions; ‘feeding site’ = sites with supplemental food (only in Italy); ‘feeding state’ = supplemental food at sites (only in Norway); ‘feeding state : to feeding state’ = transition between feeding states (only in Norway); ‘species’ = rodent species (only for Italy); ‘seasonal periods’ = seasonal variation across years; ‘sex’ = individual sex; ‘npar’ = number of parameters, ‘AICc’ = AIC with a correction for small sample sizes; ‘∆AICc’ = relative differences between the fitted model and the Akaike 'best-ranked' model with the smallest AICc value; ‘AICc weight’ = relative likelihood of a model; ‘Deviance’ = difference between null deviance and model deviance.

| **Table 5.1** | S | | | pent | | | Φ | | | p | | ψ |  |  |  |  |  |
| --- | --- | --- | --- | --- | --- | --- | --- | --- | --- | --- | --- | --- | --- | --- | --- | --- | --- |
| Model Norway | feeding state | seasonal periods | sex | feeding state | seasonal periods | session | seasonal periods | session | feeding state | session | feeding state | feeding state : to feeding state | npar | AICc | ∆AICc | AICc weight | Deviance |
| **1** | **X** | **X** |  | **X** | **X** |  |  | **X** | **X** |  | **X** | **X** | 46 | 8539.23 | 0 | 1 | 8445.99 |
| *2* | *X* | *X* | *X* | *X* |  | *X* |  | *X* | *X* | *X* | *X* | *X* | *89* | *13743.19* | *5203.96* | *0* | *13560.56* |

| **Table**  **5.2** | S | | | | | | pent | Φ | | p | | | ψ |  |  |  |  |  |
| --- | --- | --- | --- | --- | --- | --- | --- | --- | --- | --- | --- | --- | --- | --- | --- | --- | --- | --- |
| Model  Italy | feeding site | species | seasonal periods | sex | feeding site : species : sex | feeding site : species | time | species | feeding site | feeding site | session | sex | . | npar | AICc | ∆AICc | AICc  weight | Deviance |
| 1 |  | X | X | X |  |  | X | X |  | X | X |  | X | 34 | 11399.21 | 0.00 | 0.26 | 11329.41 |
| 2 |  | X | X | X |  |  | X | X |  | X | X | X | X | 35 | 11399.37 | 0.16 | 0.23 | 11327.47 |
| **3** |  |  | **X** |  | **X** |  | **X** | **X** |  | **X** | **X** |  | **X** | **40** | **11401.28** | **2.06** | **0.09** | **11318.79** |
| 4 | X | X | X | X |  |  | X | X |  | X | X |  | X | 35 | 11401.30 | 2.08 | 0.09 | 11329.39 |
| 5 | X | X | X | X |  |  | X | X |  | X | X | X | X | 36 | 11401.45 | 2.23 | 0.08 | 11327.43 |
| 6 |  |  | X |  | X |  | X | X |  | X | X | X | X | 41 | 11401.58 | 2.36 | 0.07 | 11316.96 |
| 7 |  |  | X | X |  | X | X | X |  | X | X |  | X | 37 | 11402.60 | 3.38 | 0.04 | 11326.46 |
| 8 |  |  | X | X |  |  | X | X |  | X | X | X | X | 38 | 11402.82 | 3.61 | 0.04 | 11324.57 |
| *9* |  | *X* | *X* | *X* |  |  | *X* |  | *X* | *X* | *X* |  | *X* | *34* | *11405.28* | *6.06* | *0.01* | *11335.48* |

**Appendix S6: Intra- and interspecific density-dependence**

We used the population size values at a given trapping occasion t (*N_t_*), estimated by the Multi-State Open Robust Design model, to evaluate the intra-specific density dependence of *C. glareolus* in Norway, and the intra- interspecific density dependence of *Apodemus* spp. and *C. glareolus* in Italy, where sympatry between species occurred.. Population size estimates with a value of zero were replaced with the smallest potentially observable value (0.5) (Table S6.1, Table S6.3), because populations in wild settings could not be considered entirely extinct (Huitu et al. 2003). For sites with and without supplemental food separately, we calculated population growth rate *r_t_* of rodent species for the periods between two successive primary trapping occasions t and t+1, as:

$$r_{t}=ln \left( \frac{N_{t+1}}{N_{t}} \right)$$

We then implemented regression models to assess the patterns of intraspecific and interspecific density-dependence of *C. glareolus* and *Apodemus* spp. Specifically, we modelled *C. glareolus* *r_t_* in function of (i) the ln-transformed population size of the same species (intraspecific density-dependence), both in Italy and Norway; and (ii) the ln-transformed population size of *Apodemus* spp. (interspecific density-dependence), both at sites with and without supplemental food (Table S6.2, Table S6.4). We observed a statistically significant intra-specific density dependence both in Norway and in Italy only for *C. glareolus*, both with and without supplemental feeding.

**Table S6.1**: Rounded population size estimates (N_t_) for *C. glareolus* in Norway, both with (Feeding YES) and without food (Feeding NO), for each trapping occasion t where N_t_ > 0. For *C. glareolus,* the population growth rate r_t_ at both sites is reported.

| Trapping occasion | N_t_ *C. glareolus* Feeding NO | N_t_ *C. glareolus* Feeding YES | r_t_ *C. glareolus* Feeding  NO | r_t_ *C. glareolus* Feeding YES |
| --- | --- | --- | --- | --- |
| \| 1 \| \| --- \| \| 2 \| \| 3 \| \| 4 \| \| 5 \| \| 6 \| \| 7 \| \| 8 \| \| 9 \| \| 10 \| \| 11 \| \| 12 \| \| 13 \| \| 14 \| \| 15 \| \| 16 \| \| 17 \| \| 18 \| \| 19 \| \| 20 \| \| 21 \| \| 22 \| \| 23 \| \| 24 \| | \| 102.0 \| \| --- \| \| 166.0 \| \| 125.0 \| \| 150.0 \| \| 236.0 \| \| 204.0 \| \| 0.5 \| \| 0.5 \| \| 0.5 \| \| 0.5 \| \| 0.5 \| \| 0.5 \| \| 71.0 \| \| 116.0 \| \| 151.0 \| \| 0.5 \| \| 50.0 \| \| 20.0 \| \| 17.0 \| \| 4.0 \| \| 5.0 \| \| 0.5 \| \| 5.0 \| \| 6.0 \| | \| 0.5 \| \| --- \| \| 0.5 \| \| 0.5 \| \| 0.5 \| \| 0.5 \| \| 124.0 \| \| 131.0 \| \| 146.0 \| \| 158.0 \| \| 468.0 \| \| 408.0 \| \| 330.0 \| \| 0.5 \| \| 0.5 \| \| 0.5 \| \| 286.0 \| \| 0.5 \| \| 0.5 \| \| 0.5 \| \| 0.5 \| \| 0.5 \| \| 72.0 \| \| 0.5 \| \| 0.5 \| | \| - \| \| --- \| \| 0.5 \| \| -0.3 \| \| 0.2 \| \| 0.5 \| \| -0.1 \| \| -6.0 \| \| 0.0 \| \| 0.0 \| \| 0.0 \| \| 0.0 \| \| 0.0 \| \| 4.9 \| \| 0.5 \| \| 0.3 \| \| -5.7 \| \| 4.6 \| \| -0.9 \| \| -0.2 \| \| -1.4 \| \| 0.2 \| \| -2.3 \| \| 2.3 \| \| 0.2 \| | \| - \| \| --- \| \| 0.0 \| \| 0.0 \| \| 0.0 \| \| 0.0 \| \| 5.5 \| \| 0.1 \| \| 0.1 \| \| 0.1 \| \| 1.1 \| \| -0.1 \| \| -0.2 \| \| -6.5 \| \| 0.0 \| \| 0.0 \| \| 6.3 \| \| -6.3 \| \| 0.0 \| \| 0.0 \| \| 0.0 \| \| 0.0 \| \| 5.0 \| \| -5.0 \| \| 0.0 \| |

**Table S6.2**: Regression analysis to evaluate the effect of intra-specific density dependence of *C. glareolus* in Norway, at sites with and without supplemental food (Feeding). In bold are shown the statistically significant relationships.

| Model | Feeding | Dependent variable (growth rate r_t_) | Explanatory variable (population size N_t_) | Parameter estimate (SE) | T | p-value | Model R^2^ | n |
| --- | --- | --- | --- | --- | --- | --- | --- | --- |
| 1 | YES | *C. glareolus* | Intercept | -0.84 (0.64) | -1.31 | 0.20 | 0.25 | 21 |
|  |  |  | ***C. glareolus*** | **0.51 (0.19)** | **2.68** | **0.01** |  |  |
| 2 | NO | *C. glareolus* | Intercept | -1.12 (0.61) | -1.83 | 0.08 | 0.22 | 21 |
|  |  |  | ***C. glareolus*** | **0.45 (0.19)** | **2.43** | **0.02** |  |  |

**Table S6.3**: Rounded population size estimates (N_t_) for *C. glareolus* and *Apodemus* spp., both at sites with (Feeding YES) and without food (Feeding NO), for each trapping occasion. For rodent species*,* the population growth rate r_t_ at both sites is reported.

|  | *Apodemus* spp. | | | | *C. glareolus* | | | |
| --- | --- | --- | --- | --- | --- | --- | --- | --- |
| Trapping  occasion | N_t_  Feeding NO | N_t_  Feeding YES | r_t_  Feeding  NO | r_t_  Feeding YES | N_t_  Feeding NO | N_t_  Feeding YES | r_t_  Feeding  NO | r_t_  Feeding YES |
| \| 1 \| \| --- \| \| 2 \| \| 3 \| \| 4 \| \| 5 \| \| 6 \| \| 7 \| \| 8 \| \| 9 \| \| 10 \| \| 11 \| \| 12 \| \| 13 \| \| 14 \| \| 15 \| \| 16 \| \| 17 \| \| 18 \| | \| 7 \| \| --- \| \| 13 \| \| 34 \| \| 34 \| \| 3 \| \| 2 \| \| 2 \| \| 5 \| \| 6 \| \| 8 \| \| 22 \| \| 14 \| \| 27 \| \| 22 \| \| 18 \| \| 24 \| \| 15 \| \| 28 \| | \| 6 \| \| --- \| \| 14 \| \| 84 \| \| 61 \| \| 23 \| \| 15 \| \| 8 \| \| 5 \| \| 8 \| \| 19 \| \| 42 \| \| 19 \| \| 44 \| \| 27 \| \| 46 \| \| 15 \| \| 32 \| \| 34 \| | \| NA \| \| --- \| \| 0.62 \| \| 0.96 \| \| 0.00 \| \| -2.42 \| \| -0.40 \| \| 0.00 \| \| 0.91 \| \| 0.18 \| \| 0.29 \| \| 1.01 \| \| -0.45 \| \| 0.66 \| \| -0.20 \| \| -0.20 \| \| 0.29 \| \| -0.47 \| \| 0.62 \| | \| NA \| \| --- \| \| 0.85 \| \| 1.79 \| \| -0.32 \| \| -0.98 \| \| -0.43 \| \| -0.63 \| \| -0.47 \| \| 0.47 \| \| 0.86 \| \| 0.79 \| \| -0.79 \| \| 0.84 \| \| -0.49 \| \| 0.53 \| \| -1.12 \| \| 0.76 \| \| 0.06 \| | \| 8 \| \| --- \| \| 10 \| \| 8 \| \| 7 \| \| 6 \| \| 15 \| \| 13 \| \| 15 \| \| 11 \| \| 13 \| \| 8 \| \| 6 \| \| 8 \| \| 12 \| \| 25 \| \| 14 \| \| 19 \| \| 16 \| | \| 6 \| \| --- \| \| 3 \| \| 0.5 \| \| 0.5 \| \| 2 \| \| 0.5 \| \| 2 \| \| 0.5 \| \| 3 \| \| 1 \| \| 0.5 \| \| 0.5 \| \| 0.5 \| \| 1 \| \| 0.5 \| \| 5 \| \| 16 \| \| 22 \| | \| NA \| \| --- \| \| 0.22 \| \| -0.22 \| \| -0.13 \| \| -0.15 \| \| 0.92 \| \| -0.14 \| \| 0.14 \| \| -0.31 \| \| 0.17 \| \| -0.49 \| \| -0.29 \| \| 0.29 \| \| 0.41 \| \| 0.73 \| \| -0.58 \| \| 0.31 \| \| -0.17 \| | \| NA \| \| --- \| \| -0.69 \| \| -1.78 \| \| 0.00 \| \| 1.37 \| \| -1.37 \| \| 1.37 \| \| -1.37 \| \| 1.78 \| \| -1.09 \| \| -0.68 \| \| 0.00 \| \| 0.00 \| \| 0.68 \| \| -0.68 \| \| 2.28 \| \| 1.16 \| \| 0.32 \| |

**Table S6.4**: Regression analysis to evaluate the effect of intra- and interspecific density dependence of *C. glareolus* and *Apodemus* spp. in Italy, at sites with and without supplemental food (Feeding). In bold are shown the statistically significant relationships.

| Model | Feeding | Dependent variable (growth rate r_t_) | Explanatory variable (population size N_t_) | Parameter estimate (SE) | T | p-value | Model R^2^ | n |
| --- | --- | --- | --- | --- | --- | --- | --- | --- |
| 1 | YES | *C. glareolus* | Intercept | -0.11 (0.25) | -0.44 | 0.66 | 0.36 | 15 |
|  |  |  | ***C. glareolus*** | **0.58 (0.20)** | **2.91** | **0.01** |  |  |
| 2 | NO | *C. glareolus* | Intercept | -1.19 (0.54) | -2.18 | 0.05 | 0.26 | 15 |
|  |  |  | ***C. glareolus*** | **0.51 (0.22)** | **2.29** | **0.04** |  |  |
| 3 | YES | *Apodemus* spp. | Intercept | -1.46 (0.79) | -1.86 | 0.08 | 0.22 | 15 |
|  |  |  | *Apodemus* spp. | 0.50 (0.24) | 2.05 | 0.06 |  |  |
| 4 | NO | *Apodemus* spp. | Intercept | -0.86 (0.51) | -1.66 | 0.12 | 0.20 | 15 |
|  |  |  | *Apodemus* spp. | 0.38 (0.19) | 1.05 | 0.07 |  |  |
| 5 | YES | *C. glareolus* | Intercept | 1.27 (1.27) | 1.00 | 0.33 | 0.06 | 15 |
|  |  |  | *Apodemus* spp. | -0.38 (0.39) | -0.97 | 0.35 |  |  |
| 6 | NO | *C. glareolus* | Intercept | 0.29 (0.28) | 1.05 | 0.31 | 0.06 | 15 |
|  |  |  | *Apodemus* spp. | -0.10 (0.11) | -0.97 | 0.35 |  |  |

**Appendix S7: Selected models’ output: parameters’ estimates**

**Norway – Evenstad site**

**Table S7.1**: Parameter estimates of the best model (Model1, Table S5.1), with standard error (SE) and confidence intervals (Lower, Lcl, and Upper, Ucl). Legend: ‘S’ = apparent survival; ‘pent’ = arrival probability; ‘φ’ = persistence probability; ‘p’ = capture probability; ‘ψ’ = transition probability depending on feeding states; ‘session’ = primary trapping occasions; ‘feeding state’ = supplemental food at sites; ‘seasonal periods’ = seasonal variation across years. For some sessions, the model fitting did not converge.

| Parameter | Estimate | SE | Lcl | Ucl | Feeding state | Session | Seasonal periods |
| --- | --- | --- | --- | --- | --- | --- | --- |
| S | 0.85 | 0.02 | 0.81 | 0.88 | NO | 1 | Summer2013 |
| S | 0.69 | 0.02 | 0.65 | 0.73 | NO | 5 | Winter2013-14 |
| S | 0.57 | 0.03 | 0.51 | 0.62 | NO | 10 | Summer2014 |
| S | 0.51 | 0.04 | 0.44 | 0.59 | NO | 17 | Winter2014-15 |
| S | 0.31 | 0.07 | 0.19 | 0.45 | NO | 22 | Summer2015 |
| S | 0.90 | 0.02 | 0.86 | 0.92 | YES | 1 | Summer2013 |
| S | 0.77 | 0.02 | 0.73 | 0.80 | YES | 5 | Winter2013-14 |
| S | 0.66 | 0.02 | 0.62 | 0.70 | YES | 10 | Summer2014 |
| S | 0.61 | 0.03 | 0.55 | 0.67 | YES | 17 | Winter2014-15 |
| S | 0.39 | 0.07 | 0.27 | 0.54 | YES | 22 | Summer2015 |
| pent | 0.14 | 0.00 | 0.13 | 0.14 | NO | 1 | Summer2013 |
| pent | 0.19 | 0.01 | 0.18 | 0.20 | NO | 1 | Summer2013 |
| pent | 0.45 | 0.03 | 0.39 | 0.51 | NO | 1 | Summer2013 |
| pent | 0.20 | 0.01 | 0.19 | 0.22 | YES | 1 | Winter2013-14 |
| pent | 0.26 | 0.01 | 0.23 | 0.28 | YES | 1 | Winter2013-14 |
| pent | 0.52 | 0.06 | 0.41 | 0.63 | YES | 1 | Winter2013-14 |
| phi | 0.60 | 0.05 | 0.50 | 0.70 | NO | 1 | Summer2013 |
| φ | 0.65 | 0.04 | 0.58 | 0.72 | NO | 2 | Summer2013 |
| φ | 0.70 | 0.11 | 0.47 | 0.87 | NO | 4 | Summer2013 |
| φ | 0.43 | 0.09 | 0.27 | 0.60 | YES | 6 | Winter2013-14 |
| φ | 0.44 | 0.16 | 0.18 | 0.73 | YES | 7 | Winter2013-14 |
| φ | 0.73 | 0.06 | 0.60 | 0.82 | YES | 8 | Winter2013-14 |
| φ | 0.71 | 0.06 | 0.59 | 0.80 | YES | 9 | Winter2013-14 |
| p | 0.64 | 0.02 | 0.60 | 0.68 | NO | 1 | Summer2013 |
| p | 0.43 | 0.00 | 0.43 | 0.43 | YES | 1 | Summer2013 |
| ψ | 0.85 | 0.02 | 0.82 | 0.88 | NO-NO | 1 | Summer2014 |
| ψ | 0.15 | 0.02 | 0.12 | 0.18 | NO-YES | 1 | Summer2015 |
| ψ | 0.06 | 0.01 | 0.05 | 0.08 | YES-NO | 1 | Summer2016 |
| ψ | 0.94 | 0.01 | 0.92 | 0.95 | YES-YES | 1 | Summer2017 |

**Table S7.2**: Derived estimates of population size (N_t_) retrieved by the best model (Model 1, Table S5.1), with confidence intervals (Lower, Lcl, and Upper, Ucl) and standard errors (SE). Legend: ‘session’ = primary trapping occasions; ‘feeding state’ = supplemental food at sites; ‘sex’ = individual sex, treated as group. For some sessions, the model fitting did not converge.

| Feeding state | Session | Sex | Nt | SE | Lcl | Ucl |
| --- | --- | --- | --- | --- | --- | --- |
| NO | 1.00 | Female | 51.17 | 1.34 | 48.55 | 53.79 |
| NO | 2.00 | Female | 76.99 | 1.80 | 73.46 | 80.51 |
| NO | 3.00 | Female | 65.55 | 2.28 | 61.07 | 70.03 |
| NO | 4.00 | Female | 81.22 | 2.36 | 76.60 | 85.84 |
| NO | 5.00 | Female | 117.06 | 4.08 | 109.06 | 125.06 |
| NO | 6.00 | Female | 101.45 | 3.54 | 94.52 | 108.38 |
| NO | 13.00 | Female | 29.66 | 1.03 | 27.63 | 31.68 |
| NO | 14.00 | Female | 46.82 | 1.63 | 43.62 | 50.02 |
| NO | 15.00 | Female | 81.16 | 2.83 | 75.62 | 86.71 |
| NO | 17.00 | Female | 21.85 | 0.76 | 20.36 | 23.34 |
| NO | 18.00 | Female | 7.80 | 0.27 | 7.27 | 8.34 |
| NO | 20.00 | Female | 1.56 | 0.05 | 1.45 | 1.67 |
| YES | 6.00 | Female | 63.23 | 2.71 | 57.93 | 68.54 |
| YES | 7.00 | Female | 55.93 | 2.31 | 51.41 | 60.46 |
| YES | 8.00 | Female | 72.33 | 2.16 | 68.09 | 76.57 |
| YES | 9.00 | Female | 79.71 | 2.35 | 75.10 | 84.32 |
| YES | 10.00 | Female | 213.13 | 0.00 | 213.13 | 213.13 |
| YES | 11.00 | Female | 175.66 | 0.00 | 175.66 | 175.66 |
| YES | 12.00 | Female | 145.21 | 0.00 | 145.21 | 145.21 |
| YES | 16.00 | Female | 154.58 | 0.00 | 154.58 | 154.58 |
| YES | 22.00 | Female | 36.62 | 0.23 | 36.17 | 37.08 |
| NO | 2.00 | Male | 88.54 | 2.07 | 84.48 | 92.59 |
| NO | 3.00 | Male | 59.31 | 2.07 | 55.26 | 63.36 |
| NO | 4.00 | Male | 68.61 | 1.99 | 64.71 | 72.52 |
| NO | 5.00 | Male | 118.62 | 4.13 | 110.52 | 126.72 |
| NO | 6.00 | Male | 103.01 | 3.59 | 95.97 | 110.05 |
| NO | 13.00 | Male | 40.58 | 1.41 | 37.81 | 43.35 |
| NO | 14.00 | Male | 68.67 | 2.39 | 63.98 | 73.37 |
| NO | 15.00 | Male | 70.24 | 2.45 | 65.44 | 75.03 |
| NO | 17.00 | Male | 28.09 | 0.98 | 26.17 | 30.01 |
| NO | 18.00 | Male | 12.49 | 0.44 | 11.63 | 13.34 |
| NO | 19.00 | Male | 9.36 | 0.33 | 8.72 | 10.00 |
| NO | 21.00 | Male | 4.68 | 0.16 | 4.36 | 5.00 |
| NO | 24.00 | Male | 6.24 | 0.22 | 5.82 | 6.67 |
| YES | 6.00 | Male | 61.32 | 2.63 | 56.17 | 66.46 |
| YES | 7.00 | Male | 74.58 | 3.08 | 68.55 | 80.61 |
| YES | 8.00 | Male | 73.98 | 2.21 | 69.64 | 78.31 |
| YES | 9.00 | Male | 78.05 | 2.30 | 73.53 | 82.57 |
| YES | 10.00 | Male | 255.29 | 0.00 | 255.29 | 255.29 |
| YES | 11.00 | Male | 231.87 | 0.00 | 231.87 | 231.87 |
| YES | 12.00 | Male | 185.03 | 0.00 | 185.03 | 185.03 |
| YES | 16.00 | Male | 131.16 | 0.00 | 131.16 | 131.16 |
| YES | 22.00 | Male | 35.36 | 0.22 | 34.92 | 35.80 |

**Italy – Cembra site**

**Table S7.3**: Parameter estimates of the best model (Model3, Table S5.2), with standard error (SE) and confidence intervals (Lower, Lcl, and Upper, Ucl). Legend: ‘S’ = apparent survival; ‘pent’ = arrival probability; ‘φ’ = persistence probability; ‘p’ = capture probability; ‘ψ’ = transition probability set as constant; ‘session’ = primary trapping occasions; ‘feeding site’ = sites with supplemental food; ‘species’ = rodent species; ‘seasonal periods’ = seasonal variation across years; ‘sex’ = individual sex, treated as group. For some sessions, the model fitting did not converge.

| Parameter | Estimate | SE | Lcl | Ucl | Session | Feeding site | Species | Sex |
| --- | --- | --- | --- | --- | --- | --- | --- | --- |
| S | 0.70 | 0.07 | 0.55 | 0.81 | 1 | NO | Apodemus spp. | Female |
| S | 0.45 | 0.06 | 0.34 | 0.56 | 5 | NO | Apodemus spp. | Female |
| S | 0.64 | 0.06 | 0.51 | 0.75 | 10 | NO | Apodemus spp. | Female |
| S | 0.52 | 0.06 | 0.41 | 0.63 | 15 | NO | Apodemus spp. | Female |
| S | 0.75 | 0.04 | 0.66 | 0.82 | 22 | NO | Apodemus spp. | Female |
| S | 0.78 | 0.05 | 0.66 | 0.87 | 1 | YES | Apodemus spp. | Female |
| S | 0.56 | 0.05 | 0.47 | 0.65 | 5 | YES | Apodemus spp. | Female |
| S | 0.74 | 0.05 | 0.64 | 0.82 | 10 | YES | Apodemus spp. | Female |
| S | 0.63 | 0.05 | 0.54 | 0.72 | 15 | YES | Apodemus spp. | Female |
| S | 0.82 | 0.03 | 0.75 | 0.88 | 22 | YES | Apodemus spp. | Female |
| S | 0.89 | 0.04 | 0.80 | 0.94 | 1 | NO | C. glareolus | Female |
| S | 0.74 | 0.05 | 0.62 | 0.83 | 5 | NO | C. glareolus | Female |
| S | 0.86 | 0.03 | 0.78 | 0.92 | 10 | NO | C. glareolus | Female |
| S | 0.79 | 0.05 | 0.69 | 0.87 | 15 | NO | C. glareolus | Female |
| S | 0.91 | 0.02 | 0.85 | 0.95 | 22 | NO | C. glareolus | Female |
| S | 0.64 | 0.16 | 0.30 | 0.88 | 1 | YES | C. glareolus | Female |
| S | 0.39 | 0.17 | 0.13 | 0.72 | 5 | YES | C. glareolus | Female |
| S | 0.58 | 0.18 | 0.25 | 0.85 | 10 | YES | C. glareolus | Female |
| S | 0.46 | 0.18 | 0.17 | 0.77 | 15 | YES | C. glareolus | Female |
| S | 0.70 | 0.15 | 0.37 | 0.90 | 22 | YES | C. glareolus | Female |
| S | 0.67 | 0.07 | 0.52 | 0.79 | 1 | NO | Apodemus spp. | Male |
| S | 0.42 | 0.06 | 0.31 | 0.54 | 5 | NO | Apodemus spp. | Male |
| S | 0.61 | 0.07 | 0.48 | 0.73 | 10 | NO | Apodemus spp. | Male |
| S | 0.49 | 0.06 | 0.38 | 0.61 | 15 | NO | Apodemus spp. | Male |
| S | 0.73 | 0.05 | 0.62 | 0.81 | 22 | NO | Apodemus spp. | Male |
| S | 0.62 | 0.07 | 0.46 | 0.75 | 1 | YES | Apodemus spp. | Male |
| S | 0.36 | 0.05 | 0.27 | 0.47 | 5 | YES | Apodemus spp. | Male |
| S | 0.55 | 0.07 | 0.42 | 0.68 | 10 | YES | Apodemus spp. | Male |
| S | 0.43 | 0.06 | 0.32 | 0.55 | 15 | YES | Apodemus spp. | Male |
| S | 0.68 | 0.05 | 0.57 | 0.76 | 22 | YES | Apodemus spp. | Male |
| S | 0.71 | 0.07 | 0.56 | 0.83 | 1 | NO | C. glareolus | Male |
| S | 0.47 | 0.07 | 0.34 | 0.60 | 5 | NO | C. glareolus | Male |
| S | 0.66 | 0.06 | 0.52 | 0.77 | 10 | NO | C. glareolus | Male |
| S | 0.54 | 0.07 | 0.41 | 0.66 | 15 | NO | C. glareolus | Male |
| S | 0.76 | 0.05 | 0.65 | 0.84 | 22 | NO | C. glareolus | Male |
| S | 0.66 | 0.14 | 0.37 | 0.87 | 1 | YES | C. glareolus | Male |
| S | 0.41 | 0.14 | 0.18 | 0.69 | 5 | YES | C. glareolus | Male |
| S | 0.60 | 0.14 | 0.32 | 0.83 | 10 | YES | C. glareolus | Male |
| S | 0.48 | 0.15 | 0.23 | 0.75 | 15 | YES | C. glareolus | Male |
| S | 0.72 | 0.12 | 0.45 | 0.89 | 22 | YES | C. glareolus | Male |
| pent | 0.78 | 0.04 | 0.70 | 0.84 | 1 | NO | Apodemus spp. | Female |
| pent | 0.10 | 0.04 | 0.05 | 0.20 | 1 | NO | Apodemus spp. | Female |
| pent | 0.61 | 0.07 | 0.47 | 0.73 | 2 | NO | Apodemus spp. | Female |
| pent | 0.08 | 0.03 | 0.04 | 0.16 | 2 | NO | Apodemus spp. | Female |
| pent | 0.10 | 0.07 | 0.02 | 0.35 | 2 | NO | Apodemus spp. | Female |
| pent | 0.10 | 0.07 | 0.02 | 0.34 | 2 | NO | Apodemus spp. | Female |
| pent | 0.02 | 0.04 | 0.00 | 0.65 | 2 | NO | Apodemus spp. | Female |
| φ | 0.87 | 0.03 | 0.81 | 0.92 | 1 | NO | Apodemus spp. | Female |
| φ | 0.98 | 0.02 | 0.86 | 1.00 | 1 | NO | C. glareolus | Female |
| p | 0.77 | 0.06 | 0.63 | 0.87 | 1 | NO | Apodemus spp. | Female |
| p | 0.78 | 0.05 | 0.68 | 0.86 | 2 | NO | Apodemus spp. | Female |
| p | 0.55 | 0.06 | 0.43 | 0.67 | 5 | NO | Apodemus spp. | Female |
| p | 0.71 | 0.05 | 0.61 | 0.79 | 7 | NO | Apodemus spp. | Female |
| p | 0.56 | 0.07 | 0.41 | 0.69 | 10 | NO | Apodemus spp. | Female |
| p | 0.51 | 0.07 | 0.38 | 0.65 | 11 | NO | Apodemus spp. | Female |
| p | 0.39 | 0.07 | 0.26 | 0.53 | 12 | NO | Apodemus spp. | Female |
| p | 0.56 | 0.07 | 0.43 | 0.68 | 13 | NO | Apodemus spp. | Female |
| p | 0.61 | 0.07 | 0.47 | 0.73 | 14 | NO | Apodemus spp. | Female |
| p | 0.73 | 0.06 | 0.59 | 0.83 | 15 | NO | Apodemus spp. | Female |
| p | 0.78 | 0.04 | 0.68 | 0.86 | 17 | NO | Apodemus spp. | Female |
| p | 0.71 | 0.07 | 0.57 | 0.83 | 19 | NO | Apodemus spp. | Female |
| p | 0.50 | 0.07 | 0.37 | 0.63 | 22 | NO | Apodemus spp. | Female |
| p | 0.65 | 0.05 | 0.54 | 0.74 | 23 | NO | Apodemus spp. | Female |
| p | 0.19 | 0.04 | 0.12 | 0.28 | 24 | NO | Apodemus spp. | Female |
| p | 0.53 | 0.05 | 0.42 | 0.63 | 25 | NO | Apodemus spp. | Female |
| p | 0.60 | 0.05 | 0.50 | 0.69 | 26 | NO | Apodemus spp. | Female |
| p | 0.59 | 0.05 | 0.49 | 0.69 | 27 | NO | Apodemus spp. | Female |
| p | 0.56 | 0.09 | 0.39 | 0.72 | 1 | YES | Apodemus spp. | Female |
| p | 0.57 | 0.07 | 0.43 | 0.70 | 2 | YES | Apodemus spp. | Female |
| p | 0.31 | 0.05 | 0.22 | 0.42 | 5 | YES | Apodemus spp. | Female |
| p | 0.48 | 0.06 | 0.37 | 0.59 | 7 | YES | Apodemus spp. | Female |
| p | 0.32 | 0.06 | 0.21 | 0.46 | 10 | YES | Apodemus spp. | Female |
| p | 0.28 | 0.06 | 0.18 | 0.41 | 11 | YES | Apodemus spp. | Female |
| p | 0.19 | 0.05 | 0.12 | 0.30 | 12 | YES | Apodemus spp. | Female |
| p | 0.32 | 0.06 | 0.21 | 0.45 | 13 | YES | Apodemus spp. | Female |
| p | 0.36 | 0.07 | 0.24 | 0.51 | 14 | YES | Apodemus spp. | Female |
| p | 0.50 | 0.08 | 0.35 | 0.65 | 15 | YES | Apodemus spp. | Female |
| p | 0.57 | 0.06 | 0.45 | 0.68 | 17 | YES | Apodemus spp. | Female |
| p | 0.48 | 0.08 | 0.33 | 0.64 | 19 | YES | Apodemus spp. | Female |
| p | 0.27 | 0.06 | 0.18 | 0.39 | 22 | YES | Apodemus spp. | Female |
| p | 0.40 | 0.06 | 0.30 | 0.52 | 23 | YES | Apodemus spp. | Female |
| p | 0.08 | 0.02 | 0.05 | 0.13 | 24 | YES | Apodemus spp. | Female |
| p | 0.29 | 0.05 | 0.21 | 0.39 | 25 | YES | Apodemus spp. | Female |
| p | 0.36 | 0.05 | 0.27 | 0.46 | 26 | YES | Apodemus spp. | Female |
| p | 0.35 | 0.05 | 0.26 | 0.45 | 27 | YES | Apodemus spp. | Female |

**Table S7.4**: Derived estimates of population size (N_t_) retrieved by the best model (Model3, Table S5.2) with confidence intervals (Lower, Lcl, and Upper, Ucl) and standard errors (SE). Legend: ‘session’ = primary trapping occasions; ‘feeding site’ = sites with supplemental food; ‘species’ = rodent species; ‘sex’ = individual sex, treated as group. For some sessions, the model fitting did not converge.

| Feeding site | Species | Sex | Session | Nt | SE | Lcl | Ucl |
| --- | --- | --- | --- | --- | --- | --- | --- |
| NO | Apodemus spp. | Female | 1 | 4,29 | 0,11 | 4,07 | 4,51 |
| NO | Apodemus spp. | Female | 2 | 6,33 | 0,09 | 6,15 | 6,51 |
| NO | Apodemus spp. | Female | 3 | 22,26 | 1,23 | 19,85 | 24,67 |
| NO | Apodemus spp. | Female | 4 | 14,36 | 0,34 | 13,69 | 15,03 |
| NO | Apodemus spp. | Female | 5 | 1,23 | 0,08 | 1,07 | 1,39 |
| NO | Apodemus spp. | Female | 6 | 1,28 | 0,09 | 1,10 | 1,47 |
| NO | Apodemus spp. | Female | 7 | NA | 0,00 | NA | NA |
| NO | Apodemus spp. | Female | 8 | 3,68 | 0,22 | 3,25 | 4,11 |
| NO | Apodemus spp. | Female | 9 | 2,36 | 0,12 | 2,12 | 2,61 |
| NO | Apodemus spp. | Female | 10 | 4,39 | 0,14 | 4,12 | 4,65 |
| NO | Apodemus spp. | Female | 11 | 8,55 | 0,16 | 8,24 | 8,87 |
| NO | Apodemus spp. | Female | 12 | 5,52 | 0,19 | 5,14 | 5,89 |
| NO | Apodemus spp. | Female | 13 | 18,27 | 1,38 | 15,57 | 20,96 |
| NO | Apodemus spp. | Female | 14 | 16,08 | 0,56 | 14,98 | 17,17 |
| NO | Apodemus spp. | Female | 15 | 13,33 | 2,46 | 8,51 | 18,15 |
| NO | Apodemus spp. | Female | 16 | 13,94 | 0,75 | 12,47 | 15,41 |
| NO | Apodemus spp. | Female | 17 | 10,68 | 0,41 | 9,88 | 11,48 |
| NO | Apodemus spp. | Female | 18 | 15,54 | 0,64 | 14,29 | 16,78 |
| YES | Apodemus spp. | Female | 1 | 2,45 | 0,19 | 2,08 | 2,82 |
| YES | Apodemus spp. | Female | 2 | 4,59 | 0,17 | 4,25 | 4,93 |
| YES | Apodemus spp. | Female | 3 | 40,46 | 4,69 | 31,26 | 49,66 |
| YES | Apodemus spp. | Female | 4 | 29,37 | 1,87 | 25,70 | 33,03 |
| YES | Apodemus spp. | Female | 5 | 13,91 | 2,00 | 10,00 | 17,83 |
| YES | Apodemus spp. | Female | 6 | 11,41 | 1,76 | 7,96 | 14,85 |
| YES | Apodemus spp. | Female | 7 | 5,18 | 1,06 | 3,10 | 7,26 |
| YES | Apodemus spp. | Female | 8 | 5,19 | 0,72 | 3,78 | 6,60 |
| YES | Apodemus spp. | Female | 9 | 6,34 | 0,80 | 4,77 | 7,92 |
| YES | Apodemus spp. | Female | 10 | 14,38 | 1,25 | 11,93 | 16,83 |
| YES | Apodemus spp. | Female | 11 | 20,69 | 1,07 | 18,59 | 22,78 |
| YES | Apodemus spp. | Female | 12 | 10,65 | 1,03 | 8,64 | 12,65 |
| YES | Apodemus spp. | Female | 13 | 19,67 | 3,05 | 13,70 | 25,64 |
| YES | Apodemus spp. | Female | 14 | 11,83 | 1,05 | 9,78 | 13,88 |
| YES | Apodemus spp. | Female | 15 | 17,34 | 4,21 | 9,08 | 25,59 |
| YES | Apodemus spp. | Female | 16 | 3,71 | 0,44 | 2,85 | 4,57 |
| YES | Apodemus spp. | Female | 17 | 19,23 | 1,73 | 15,84 | 22,62 |
| YES | Apodemus spp. | Female | 18 | 21,18 | 1,97 | 17,31 | 25,04 |
| NO | C. glareolus | Female | 1 | 3,14 | 0,06 | 3,02 | 3,27 |
| NO | C. glareolus | Female | 2 | 5,14 | 0,04 | 5,06 | 5,23 |
| NO | C. glareolus | Female | 3 | 3,54 | 0,18 | 3,19 | 3,88 |
| NO | C. glareolus | Female | 4 | 3,22 | 0,06 | 3,09 | 3,34 |
| NO | C. glareolus | Female | 5 | 4,70 | 0,27 | 4,16 | 5,24 |
| NO | C. glareolus | Female | 6 | 10,97 | 0,71 | 9,59 | 12,36 |
| NO | C. glareolus | Female | 7 | 9,96 | 1,09 | 7,83 | 12,09 |
| NO | C. glareolus | Female | 8 | 9,38 | 0,49 | 8,41 | 10,34 |
| NO | C. glareolus | Female | 9 | 5,67 | 0,26 | 5,16 | 6,17 |
| NO | C. glareolus | Female | 10 | 6,39 | 0,16 | 6,09 | 6,70 |
| NO | C. glareolus | Female | 11 | 5,23 | 0,08 | 5,08 | 5,38 |
| NO | C. glareolus | Female | 12 | 1,07 | 0,03 | 1,01 | 1,13 |
| NO | C. glareolus | Female | 13 | 2,48 | 0,17 | 2,14 | 2,81 |
| NO | C. glareolus | Female | 14 | 5,53 | 0,16 | 5,21 | 5,85 |
| NO | C. glareolus | Female | 15 | 9,83 | 1,78 | 6,34 | 13,32 |
| NO | C. glareolus | Female | 16 | 7,24 | 0,36 | 6,54 | 7,93 |
| NO | C. glareolus | Female | 17 | 7,97 | 0,26 | 7,45 | 8,48 |
| NO | C. glareolus | Female | 18 | 9,16 | 0,33 | 8,52 | 9,80 |
| YES | C. glareolus | Female | 1 | 3,51 | 0,24 | 3,04 | 3,99 |
| YES | C. glareolus | Female | 2 | 1,08 | 0,03 | 1,03 | 1,13 |
| YES | C. glareolus | Female | 10 | 1,24 | 0,10 | 1,05 | 1,43 |
| YES | C. glareolus | Female | 17 | 9,00 | 0,78 | 7,47 | 10,52 |
| YES | C. glareolus | Female | 18 | 7,62 | 0,69 | 6,27 | 8,96 |
| NO | Apodemus spp. | Male | 1 | 3,22 | 0,08 | 3,05 | 3,38 |
| NO | Apodemus spp. | Male | 2 | 7,39 | 0,11 | 7,17 | 7,60 |
| NO | Apodemus spp. | Male | 3 | 12,37 | 0,68 | 11,03 | 13,71 |
| NO | Apodemus spp. | Male | 4 | 19,88 | 0,47 | 18,95 | 20,81 |
| NO | Apodemus spp. | Male | 5 | 2,46 | 0,16 | 2,14 | 2,78 |
| NO | Apodemus spp. | Male | 7 | 1,52 | 0,18 | 1,17 | 1,86 |
| NO | Apodemus spp. | Male | 8 | 1,23 | 0,07 | 1,08 | 1,37 |
| NO | Apodemus spp. | Male | 9 | 3,54 | 0,19 | 3,18 | 3,91 |
| NO | Apodemus spp. | Male | 11 | 12,83 | 0,24 | 12,36 | 13,30 |
| NO | Apodemus spp. | Male | 12 | 7,72 | 0,27 | 7,19 | 8,25 |
| NO | Apodemus spp. | Male | 13 | 9,13 | 0,69 | 7,78 | 10,48 |
| NO | Apodemus spp. | Male | 14 | 5,74 | 0,20 | 5,35 | 6,13 |
| NO | Apodemus spp. | Male | 15 | 5,33 | 0,98 | 3,41 | 7,26 |
| NO | Apodemus spp. | Male | 16 | 10,14 | 0,55 | 9,07 | 11,21 |
| NO | Apodemus spp. | Male | 17 | 3,56 | 0,14 | 3,29 | 3,83 |
| NO | Apodemus spp. | Male | 18 | 11,95 | 0,49 | 10,99 | 12,91 |
| YES | Apodemus spp. | Male | 1 | 3,68 | 0,28 | 3,12 | 4,24 |
| YES | Apodemus spp. | Male | 2 | 9,18 | 0,35 | 8,50 | 9,86 |
| YES | Apodemus spp. | Male | 3 | 43,98 | 5,10 | 33,98 | 53,98 |
| YES | Apodemus spp. | Male | 4 | 32,04 | 2,04 | 28,04 | 36,03 |
| YES | Apodemus spp. | Male | 5 | 8,70 | 1,25 | 6,25 | 11,14 |
| YES | Apodemus spp. | Male | 6 | 3,80 | 0,59 | 2,65 | 4,95 |
| YES | Apodemus spp. | Male | 7 | 2,59 | 0,53 | 1,55 | 3,63 |
| YES | Apodemus spp. | Male | 9 | 1,59 | 0,20 | 1,19 | 1,98 |
| YES | Apodemus spp. | Male | 10 | 5,23 | 0,45 | 4,34 | 6,12 |
| YES | Apodemus spp. | Male | 12 | 7,98 | 0,77 | 6,48 | 9,49 |
| YES | Apodemus spp. | Male | 13 | 23,61 | 3,65 | 16,44 | 30,77 |
| YES | Apodemus spp. | Male | 14 | 14,79 | 1,31 | 12,23 | 17,35 |
| YES | Apodemus spp. | Male | 15 | 28,89 | 7,02 | 15,13 | 42,66 |
| YES | Apodemus spp. | Male | 16 | 11,13 | 1,31 | 8,55 | 13,70 |
| YES | Apodemus spp. | Male | 17 | 12,82 | 1,15 | 10,56 | 15,08 |
| YES | Apodemus spp. | Male | 18 | 13,03 | 1,21 | 10,66 | 15,41 |
| NO | C. glareolus | Male | 1 | 5,24 | 0,11 | 5,03 | 5,44 |
| NO | C. glareolus | Male | 4 | 4,29 | 0,08 | 4,12 | 4,45 |
| NO | C. glareolus | Male | 5 | 1,17 | 0,07 | 1,04 | 1,31 |
| NO | C. glareolus | Male | 6 | 3,66 | 0,24 | 3,20 | 4,12 |
| NO | C. glareolus | Male | 7 | 2,85 | 0,31 | 2,24 | 3,45 |
| NO | C. glareolus | Male | 8 | 5,86 | 0,31 | 5,25 | 6,47 |
| NO | C. glareolus | Male | 9 | 4,53 | 0,21 | 4,13 | 4,94 |
| NO | C. glareolus | Male | 10 | 7,46 | 0,18 | 7,10 | 7,82 |
| NO | C. glareolus | Male | 11 | 3,14 | 0,05 | 3,05 | 3,23 |
| NO | C. glareolus | Male | 12 | 5,36 | 0,15 | 5,06 | 5,65 |
| NO | C. glareolus | Male | 13 | 6,19 | 0,42 | 5,36 | 7,02 |
| NO | C. glareolus | Male | 15 | 14,74 | 2,67 | 9,50 | 19,97 |
| NO | C. glareolus | Male | 17 | 11,38 | 0,38 | 10,64 | 12,12 |
| NO | C. glareolus | Male | 18 | 6,87 | 0,25 | 6,39 | 7,35 |
| YES | C. glareolus | Male | 1 | 2,34 | 0,16 | 2,03 | 2,66 |
| YES | C. glareolus | Male | 2 | 2,16 | 0,05 | 2,06 | 2,26 |
| YES | C. glareolus | Male | 5 | 1,62 | 0,22 | 1,18 | 2,06 |
| YES | C. glareolus | Male | 7 | 2,39 | 0,48 | 1,45 | 3,33 |
| YES | C. glareolus | Male | 9 | 2,97 | 0,36 | 2,26 | 3,68 |
| YES | C. glareolus | Male | 14 | 1,39 | 0,12 | 1,16 | 1,62 |
| YES | C. glareolus | Male | 16 | 5,17 | 0,60 | 4,00 | 6,35 |
| YES | C. glareolus | Male | 17 | 7,50 | 0,65 | 6,23 | 8,77 |
| YES | C. glareolus | Male | 18 | 13,71 | 1,23 | 11,29 | 16,13 |

**Appendix S8: Ancillary demographic parameters**

*Persistence probability*

Persistence probability (*φ*) showed differences across the two study areas. In particular, in Norway *φ* in *C. glareolus* varied through sessions with slight differences between winter and summer, but no feeding effect was detected (Fig. S8.1).

In Italy, on the contrary, *φ* was affected by species and it was higher in *C. glareolus* than in *Apodemus spp.* (Fig. S8.1).


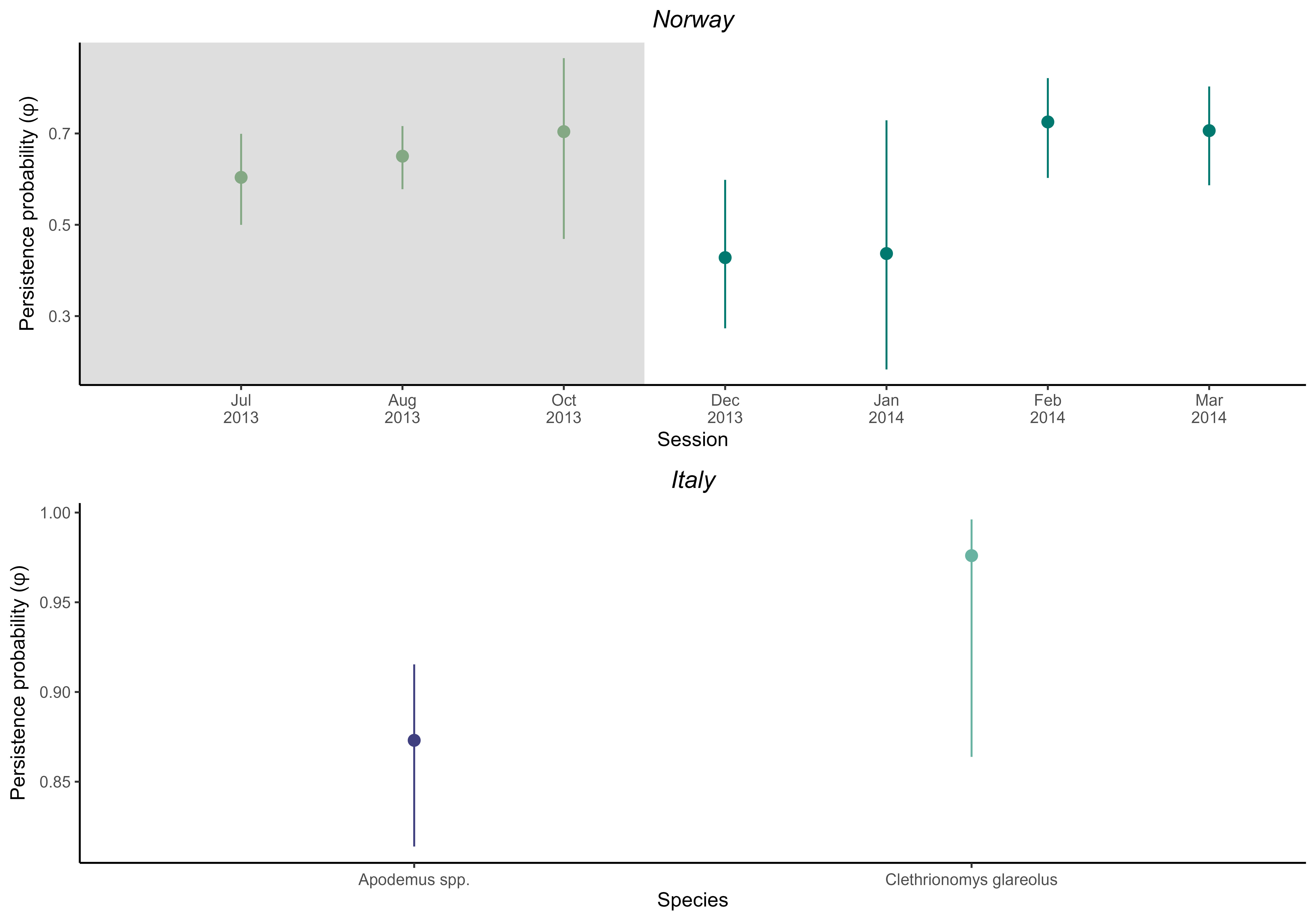


**Figure S8.1**: Real estimates of persistence probability (φ) in Norway (upper panel) depending on seasonal periods, and under different food availability condition (supplemental food available = dark green; no supplemental food available = light green); while in Italy (lower panel) for each detected species (*C. glareolus* and *Apodemus spp.*). Grey shaded bars indicate the summer periods, while white bars denote the winter periods.

*Arrival probability*

Arrival probability (*pent*) was time-dependent both in Norway and in Italy. In Norway *pent* retained a temporal effect which depended on seasonal periods (Fig. S8.2). In particular, *pent* was particularly high during autumn when food was not provided.

In Italy *pent* was affected by secondary trapping occasions, with higher values at the first secondary occasion, which decreased with the subsequent occasions (Fig. S8.2).


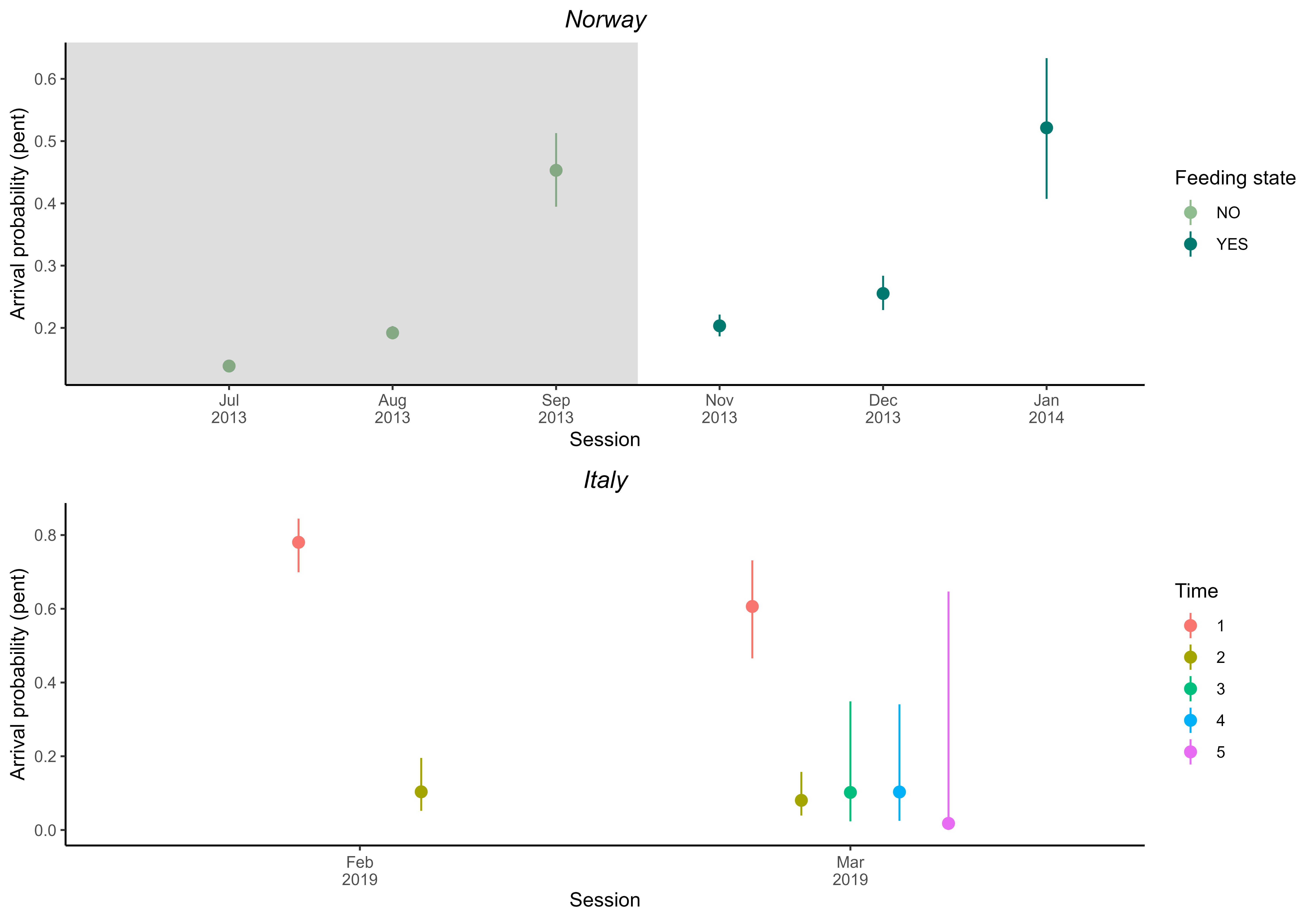


**Figure S8.2**: Real estimates of arrival probability (*pent*) in Norway (upper panel) for each primary trapping occasion and under different food availability condition (supplemental food available = dark green; no supplemental food available = light green), and in Italy (lower panel) for each secondary trapping occasion. Grey shaded bars indicate the summer periods, while white bars denote the winter periods. The secondary occasions when the model fitting did not converge are not reported.

*Capture probability*

In Norway, *p* depended only on feeding state and in particular, when supplemental food was provided, animals were less likely to be captured than without food (Fig. S8.3).

Similarly, in Italy *p* changed in relation with supplemental food and across primary occasions. In particular, *p* was lower where food was provided during the entire sampling period for both species. In general, *p* declined from summer to winter, with some exceptions (December 2020 and January 2021) (Fig. S8.3).


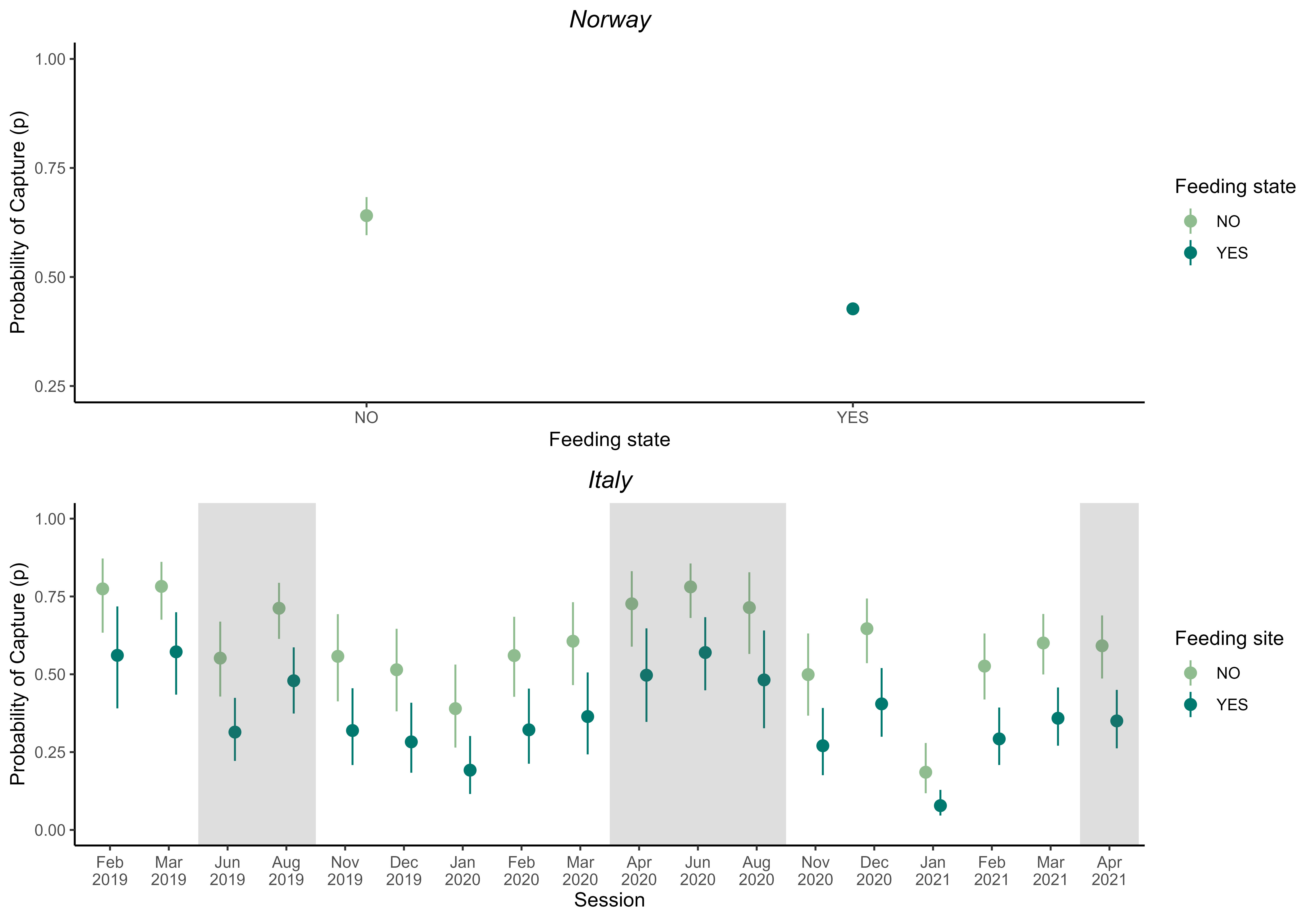


**Figure S8.3**: Real estimates of capture probability (*p*) in Norway (upper panel) and in Italy (lower panel) for each primary trapping occasion and under different food availability conditions (supplemental food available = dark green; no supplemental food available = light green). Grey shaded bars indicate the summer periods, while white bars denote the winter periods.

**Appendix S9: Residence time**


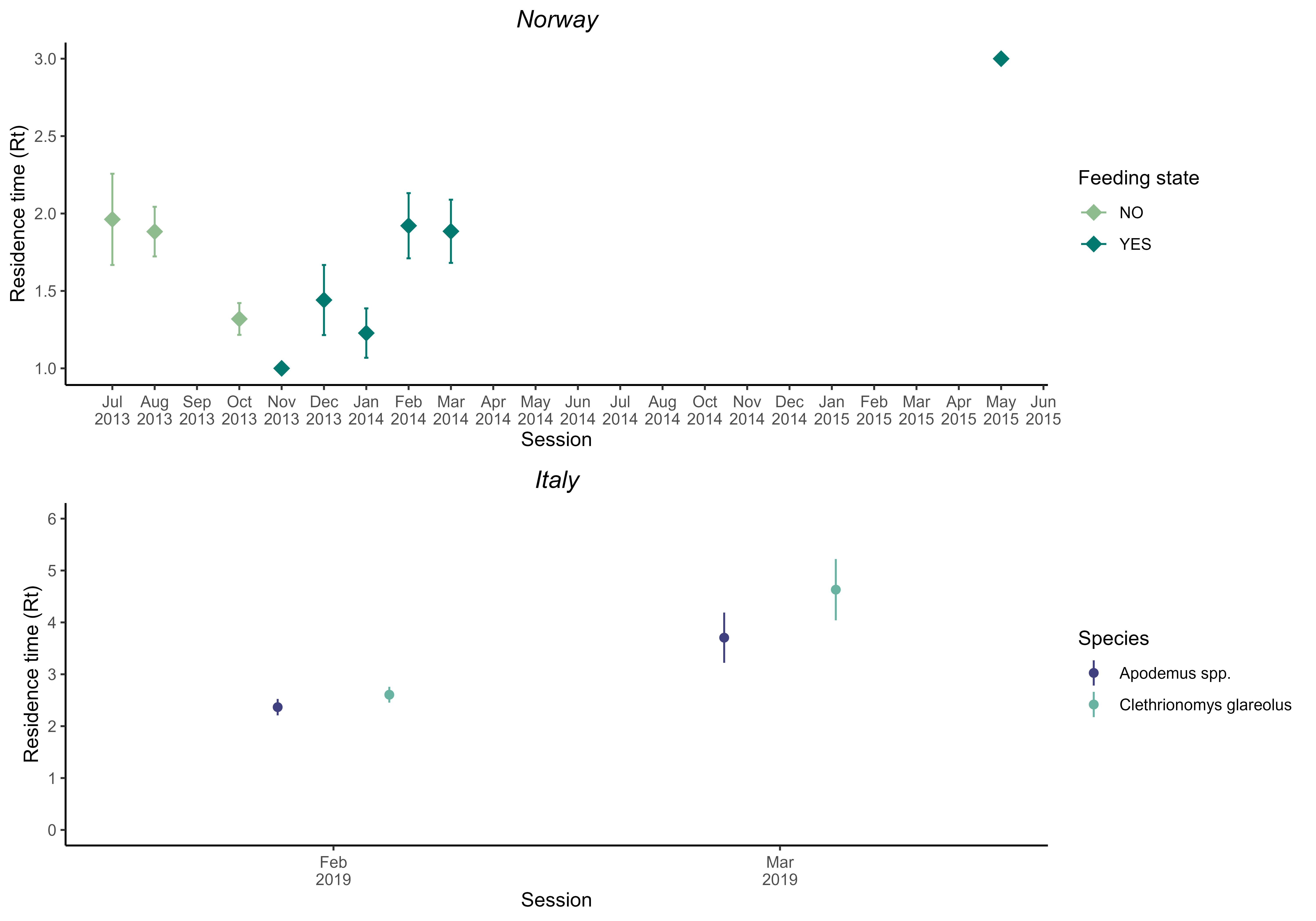


**Figure S9**: Derived estimates of residence time (R_t_) in Norway (upper panel) under different food availability conditions (supplemental food available = dark green; no supplemental food available = light green) and for each primary trapping occasion, and in Italy (lower panel) for each detected species (*C. glareolus* and *Apodemus spp.*), for each primary trapping occasion.

**References**

Burnham, Kenneth P., and David R. Anderson. 2002. “A Practical Information-Theoretic Approach.” In *Model Selection and Multimodel Inference*, 2nd edn., 70–71. Springer-Verlag, New York, NY, USA.

Huitu, Otso, Minna Koivula, Erkki Korpimäki, Tero Klemola, and Kai Norrdahl. 2003. “Winter Food Supply Limits Growth of Northern Vole Populations in the Absence of Predation.” *Ecology* 84 (8): 2108–18. https://doi.org/10.1890/02-0040.

Laake, JL Jeff, and Eric Rexstad. 2008. “RMark—an Alternative Approach to Building Linear Models. Appendix C.” In *Program MARK: A Gentle Introduction.*, C1–111.
